# Author Correction: Deep brain stimulation of symptom-specific networks in Parkinson’s disease

**DOI:** 10.1038/s41467-024-51394-7

**Published:** 2024-08-20

**Authors:** Nanditha Rajamani, Helen Friedrich, Konstantin Butenko, Till Dembek, Florian Lange, Pavel Navrátil, Patricia Zvarova, Barbara Hollunder, Rob M. A. de Bie, Vincent J. J. Odekerken, Jens Volkmann, Xin Xu, Zhipei Ling, Chen Yao, Petra Ritter, Wolf-Julian Neumann, Georgios P. Skandalakis, Spyridon Komaitis, Aristotelis Kalyvas, Christos Koutsarnakis, George Stranjalis, Michael Barbe, Vanessa Milanese, Michael D. Fox, Andrea A. Kühn, Erik Middlebrooks, Ningfei Li, Martin Reich, Clemens Neudorfer, Andreas Horn

**Affiliations:** 1 Movement Disorder and Neuromodulation Unit, Department of Neurology, Charité-Universitätsmedizin Berlin, corporate member of Freie Universität Berlin and Humboldt-Universität zu Berlin, Berlin, Germany; 2 Center for Brain Circuit Therapeutics Department of Neurology Brigham & Women’s Hospital, Harvard Medical School, Boston, MA USA; 3 University of Würzburg, Faculty of Medicine, Josef-Schneider-Str. 2, 97080 Würzburg, Germany; 4 Department of Neurology, University of Cologne, Cologne, Germany; 5 Department of Neurology, University Clinic of Würzburg, Josef-Schneider-Str. 11, 97080 Würzburg, Germany; 6 Einstein Center Digital Future, Berlin, 10117 Germany; 7 Brain Simulation Section, Department of Neurology, Charité University Medicine Berlin and Berlin Institute of Health, Berlin, 10117 Germany; 8 Department of Neurology, Amsterdam University Medical Center, Amsterdam, The Netherlands; 9 Department of Neurosurgery, Chinese PLA General Hospital, Beijing, 100853 China; 10 Department of Neurosurgery, Hainan Hospital of Chinese PLA General Hospital, Sanya, Hainan 572000 China; 11 Department of Neurosurgery, The National Key Clinic Specialty, Shenzhen Key Laboratory of Neurosurgery, the First Affiliated Hospital of Shenzhen University, Shenzhen Second People’s Hospital, Shenzhen, 518035 China; 12 Bernstein center for Computational Neuroscience Berlin, Berlin, 10117 Germany; 13 Section of Neurosurgery, Dartmouth Hitchcock Medical Center, Lebanon, NH 03756 USA; 14 Department of Neurosurgery, National and Kapodistrian University of Athens Medical School, Evangelismos General Hospital, Athens, Greece; 15 Centre for Spinal Studies and Surgery, Queen’s Medical Centre, Nottingham University Hospitals NHS Trust, Nottingham, UK; 16 Division of Neurosurgery, Toronto Western Hospital, University Health Network, Toronto, ON Canada; 17 Neurosurgical Division, Hospital Beneficência Portuguesa de São Paulo, São Paulo, Brazil; 18 Department of Neurosurgery, Mayo Clinic, Florida, USA; 19 Movement Disorders and Neuromodulation Unit, DOMMO Clinic, São Paulo, Brazil; 20 Harvard Medical School, Boston, MA 02114 USA; 21 Brain Modulation Lab, Department of Neurosurgery, Massachusetts General Hospital, Boston, MA 02114 USA; 22 Department of Radiology, Mayo Clinic Florida, Jacksonville, FL USA

**Keywords:** Basal ganglia, Parkinson's disease, Network models

Correction to: *Nature Communications* 10.1038/s41467-024-48731-1, published online 31 May 2024

The original version of this Article contained an error in Figure 2, in which the statistical analyses in the insets of panel B were incorrectly reported. Permutation tests were inadvertently reported instead of circular tests and cross-validation values.

The correct version of Figure 2 is:
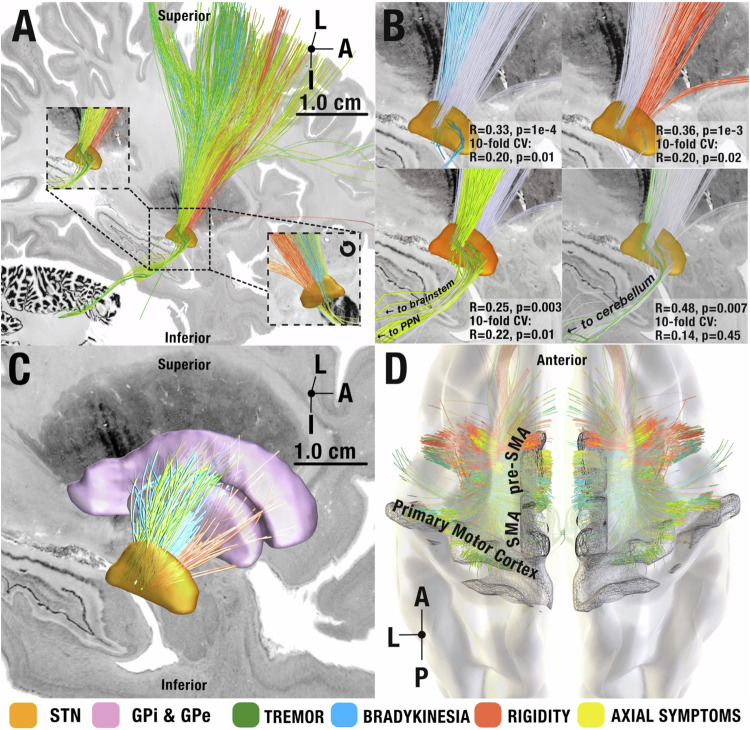


Which replaces the previous incorrect version:
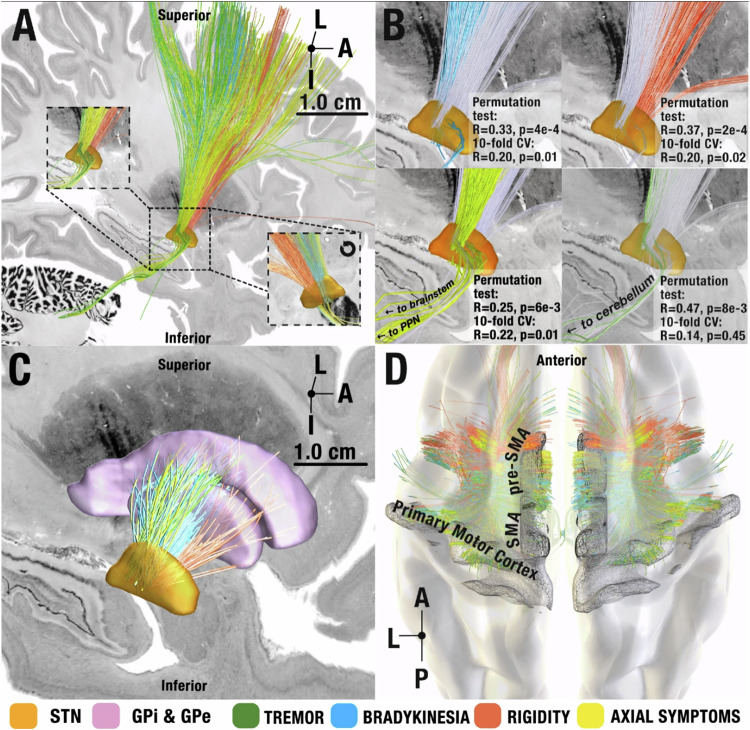


The original version of this Article contained an error in the text in the third paragraph of the results section ‘Symptom-Response Multi-Tract Model (Discovery Cohort)’ on page 3, which incorrectly read ‘Here, all symptom tracts significantly explained more variance in outcomes than re-calculated tract models after permuting improvement values across patients 1000 times (p < 0.001). Second, we subjected tract models to cross-validations. Here, all but the tremor tract model explained statistically significant amounts of variance when subjected to 10-fold cross-validations (bradykinesia: R = 0.20, p = 0.02; rigidity R = 0.20, p = 0.02; axial symptoms R = 0.22, p = 0.01, also see Fig. 2)’.

The correct version states ‘Here, the bradykinesia and rigidity tracts significantly explained more variance in outcomes than re-calculated tract models after permuting improvement values across patients 1,000 times (p < 0.05). Second, we subjected tract models to cross-validations. Here, all but the tremor tract model explained statistically significant amounts of variance when subjected to 10-fold cross-validations (bradykinesia: R = 0.20, p = 0.02; rigidity R = 0.20, p = 0.02; axial symptoms R = 0.22, p = 0.01, also see Fig. 2)’.

The original version of this Article contained an error in the text in the figure legend of Figure 2 for panel B, which incorrectly read ‘B Symptom-response tracts visualized separately at the STN level with the other tracts grayed out for spatial comparison. Insets represent permutation tests and 10-fold cross-validation results for each symptom tract’.

The correct version states ‘B Symptom-response tracts visualized separately at the STN level with the other tracts grayed out for spatial comparison. Insets represent circular and 10-fold cross-validation results for each symptom tract’.

This has been corrected in both the PDF and HTML versions of the Article.

