# Supplementary material for: Deep brain stimulation of symptom-specific networks in Parkinson’s disease
Source: Nat Commun. 2024 May 31;15:4662. doi: 10.1038/s41467-024-48731-1 (PMC11143329; doi:10.1038/s41467-024-48731-1)
Supplement: Supplementary file 1 — Supplementary Information [file 41467_2024_48731_MOESM1_ESM.pdf]

Deep Brain Stimulation of Symptom-Specific Networks in Parkinson's Disease
(Rajamani et al., 2024)

Supplementary Information

Supplementary Discussion

S1. Variability in Electrode Placement

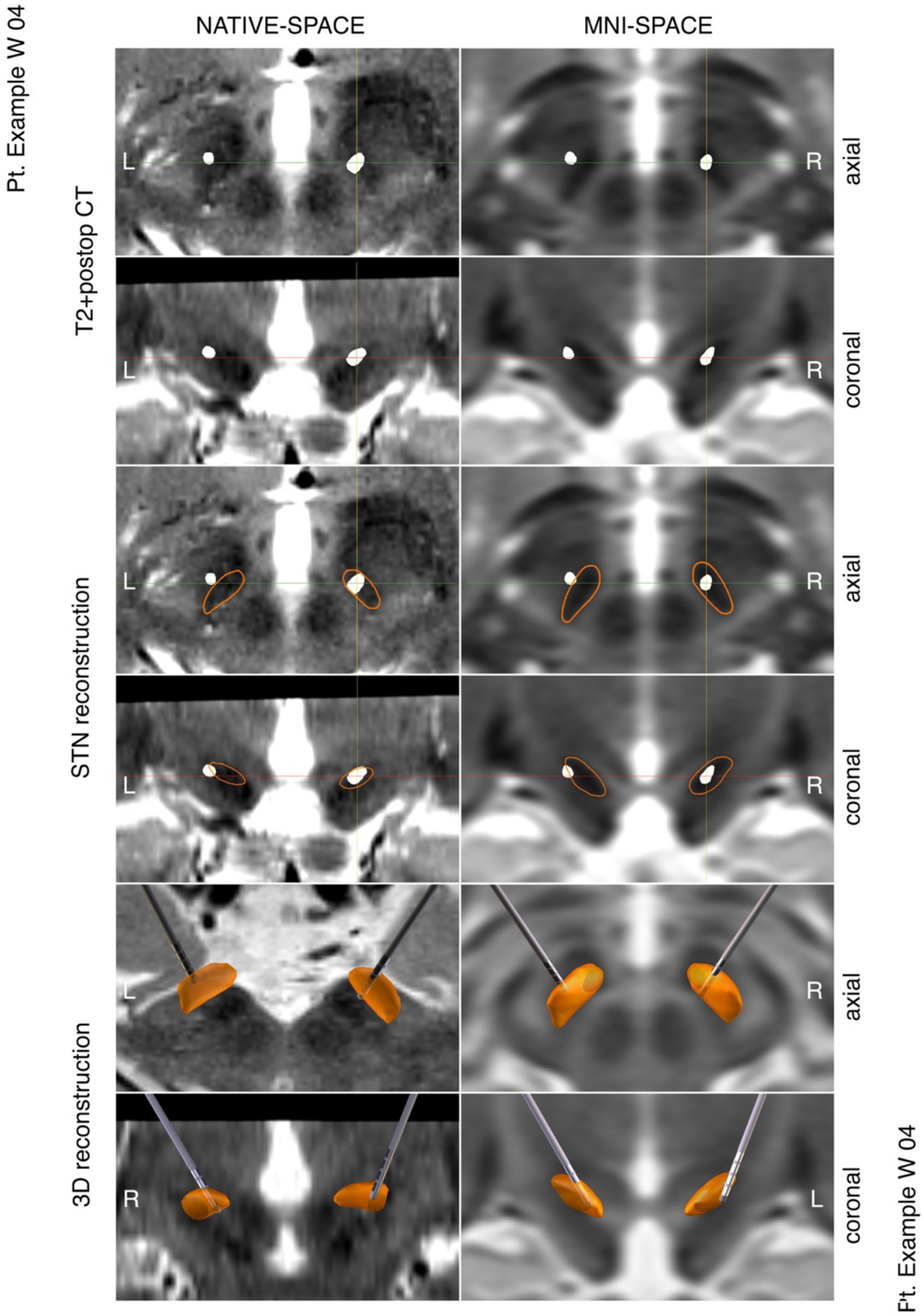

Figure S1. Comparison of imaging data with 3D DBS models of example cases with suboptimal electrode placements (continued on the next pages). Each figure shows (in order from top to bottom): A preoperative T2 (axial & coronal sections) with thresholded registered postoperative CT superimposed for the cases of postoperative CT usage, the same views superimposing the segmented subthalamic nucleus (based on the

diffeomorphic transform following spatial normalization and WarpDrive correction), and a 3D reconstruction of the same data (Lead-DBS output). The left columns shows the respective views in native (AC/PC registered) space, the right columns show the same data after transform into MNI space.

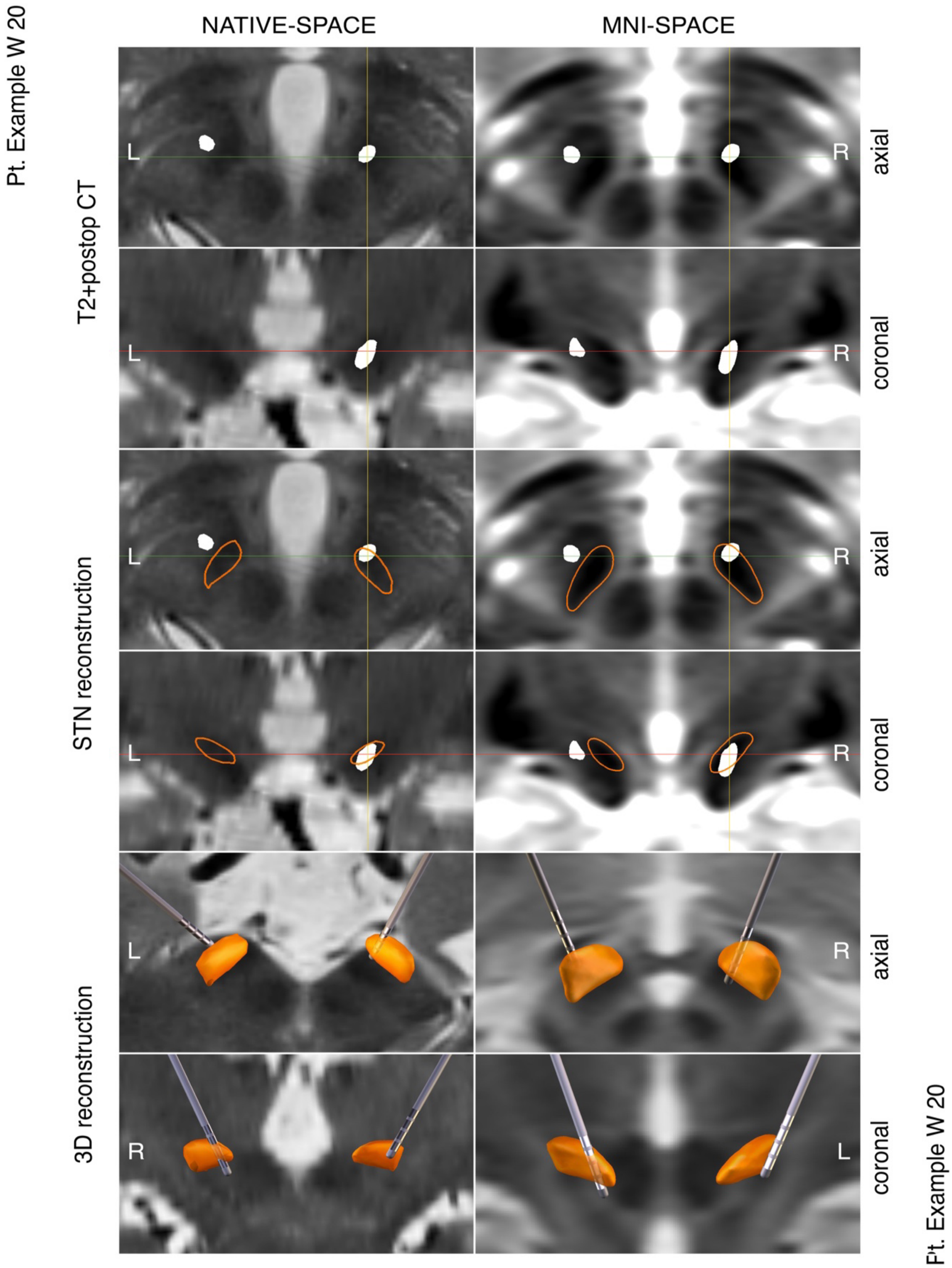

Figure S1 (continued).

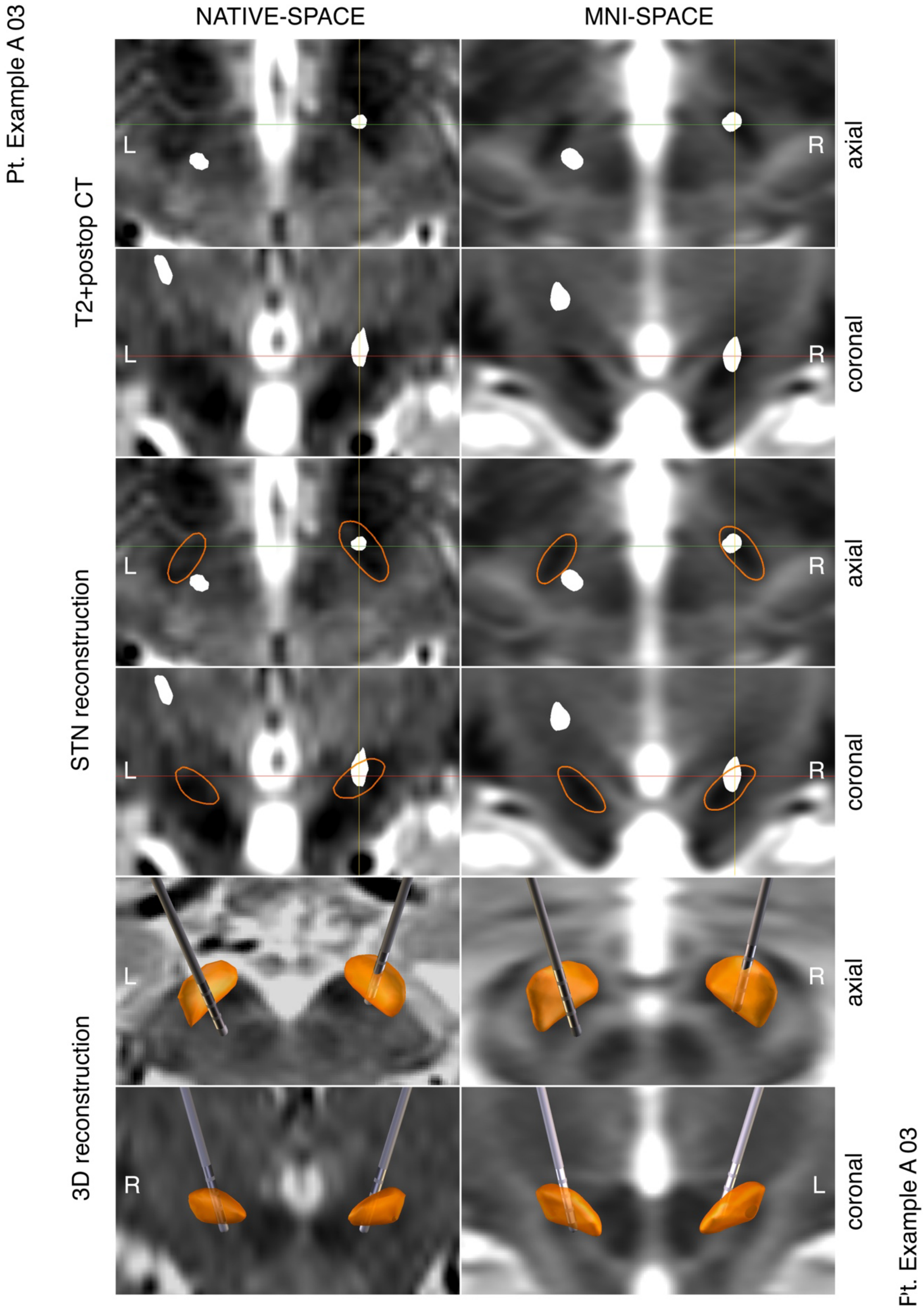

Figure S1 (continued).

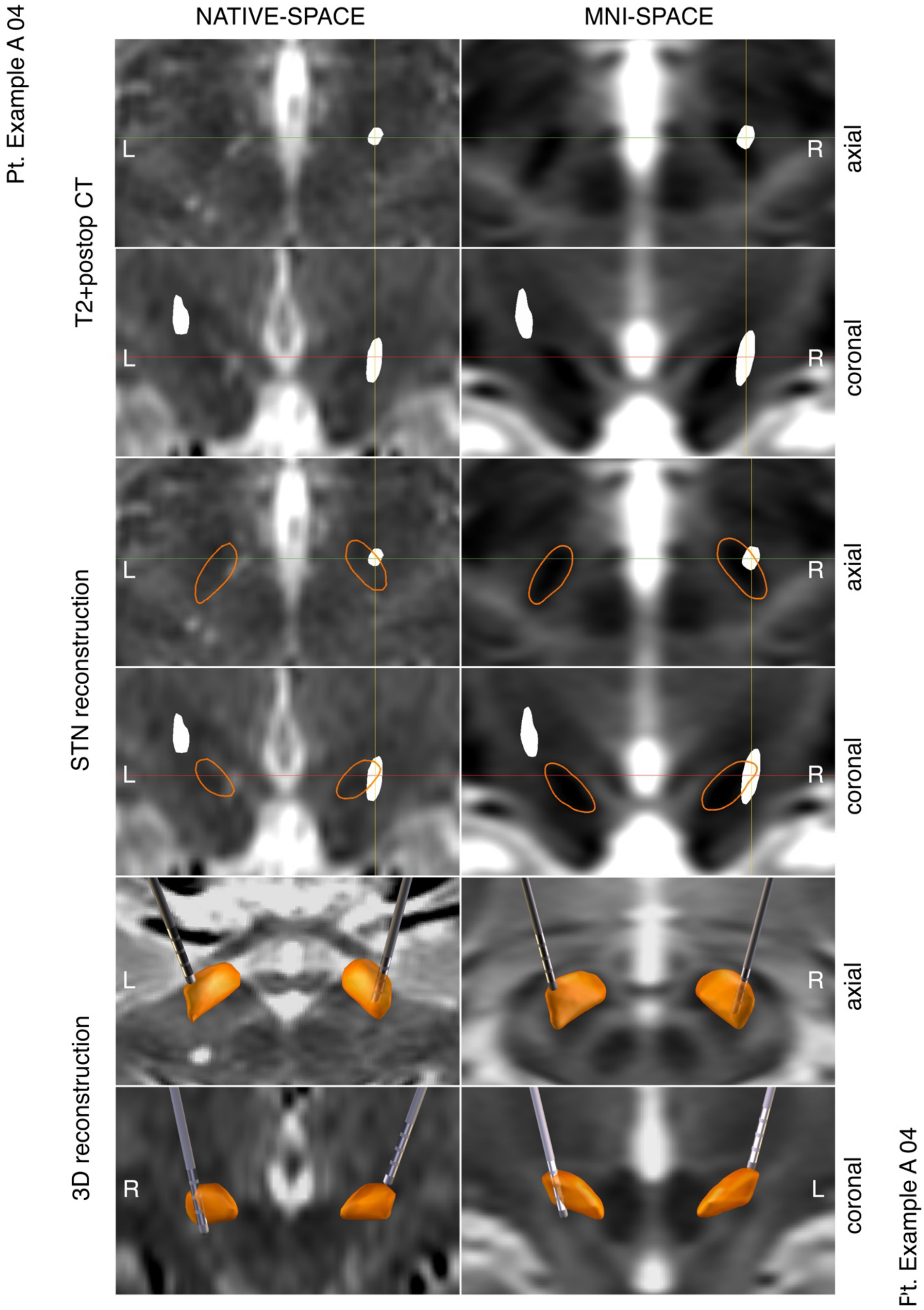

Figure S1 (continued).

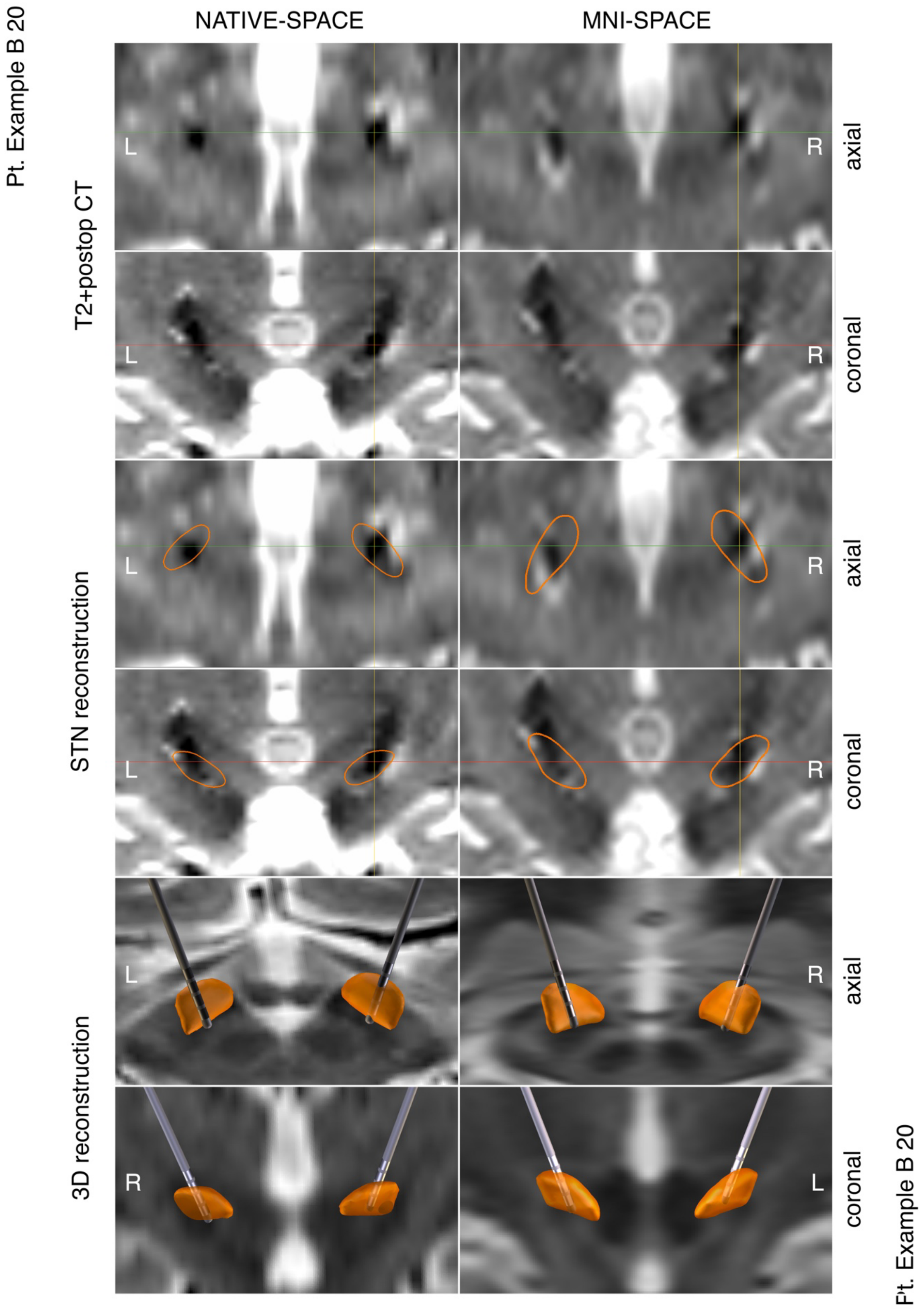

Figure S1 (continued).

S2. Validation in Support of Lead-DBS methodology

Table S1: Published reports in support of Lead-DBS methodology.

The table outlines studies that demonstrated the utility of the Lead-DBS toolbox, each validating specific aspects (or multiple aspects) of its processing pipeline. Critically, some of these validations are of indirect measure (such as estimates of clinical outcomes in unseen datasets). However, others are directly targeted to validate specific components of the pipeline (such as interrater and inter-modality comparisons of electrode placements or direct comparisons of LFP and imaging derived definitions of subcortical nuclei). In their sum, these studies may demonstrate accuracy and utility of the Lead-DBS toolbox. Abbreviations: CT: Computed Tomography, STN: subthalamic nucleus, GPi: internal pallidum, MER: Microelectrode recordings, MUA: Multiunit activity, VTA: volumes of tissue activated, PD: Parkinson's disease, DYT: dystonia.

Validated Concept	Validated using…	Study	Notes
Electrode Localizations	Phantom Validations	1	This study validated the automatic electrode detection algorithm applied in Lead-DBS using phantom data scanned in the CT.
	Test-Retest / Inter-rater agreement	2	The study compared electrode localizations carried out by six raters after minimal training, showing an average difference in localizations between 0.52–0.75 mm.
	LFP-Recordings	3–5	These studies showed that peak beta-power magnitudes (and other LFP markers) localized to a common site within the STN across PD patients, which requires millimeter precision of electrode localizations.
		6	This study showed that theta-power magnitudes localized to a specific site within the GPi across DYT patients, which requires millimeter precision of electrode localizations.
		7	These studies showed that gamma-power magnitudes localized to a common site within the STN across PD patients that executed a movement task, which requires millimeter precision of electrode localizations.

Validated Concept	Validated using…	Study	Notes
		8	This study showed that high-spatial-resolution STN microelectrode electrophysiology recordings of PD patients (933 electrode trajectories) matched DBS electrode localizations obtained with Lead-DBS using imaging data.
		9	This study evidenced a strong accuracy in the position of electrode localized with Lead DBS (postoperative image reconstruction) and anatomical locations of intraoperative individual MERs (231 MERs, 144 in 34 STNs, 7 in 4 thalami, 5 in 4 ZIs, 34 in 10 SNs, 41 others) with an average difference in depth of the dorsal STN entry of 0.1 mm (standard deviation: 0.8 mm).
		10	This study showed the concordance between probabilistic electrode locations using Lead-DBS and intraoperative local field potential recordings in PD patients implanted in the STN with Vercise Cartesia directional electrodes (Boston Scientific).
	CT/MRI comparisons	712,11,12	These studies showed highly comparable results when localizing electrodes based on postoperative CTs vs. MRIs.
	Comparison to other software	12	This study compared DBS electrode reconstruction performed with Lead-DBS and Surgiplan and showed no significant difference in the relative distance of the electrode and the STN between the two methods (around 1mm coordinate difference).
Directionality Detection	Phantom validations	13–15	These studies extensively validated the DiODe algorithm used for directionality detection using phantom and clinical datasets.
	Temporal Stability / Test-Retest Estimates	16	This study analyzed the temporal stability of directional DBS lead orientation using the DiODe algorithm implemented in Lead-DBS for 29 leads at 48 timepoints (up to 811 days). The mean difference of the orientation angles compared to the initial measurement was –1.1 ± 3.9° (no significant difference), showing the constancy of the model over time and indirectly showing test-retest comparability of DiODe.

Validated Concept	Validated using…	Study	Notes
Bioelectric Modeling	LFP-Recordings	[17]	The study showed a significant inverse correlation between % of the subthalamic nucleus stimulated (as modeled by Lead-DBS) and the %-change in beta burst durations.
	Clinical observations	[18]	This study showed a clear mapping between modeled VTAs and subsequent tract activations (using both normative and patient-specific tractography) and (side-) effect thresholds.
	Comparison to alternate software	[19]	This study compares bioelectrical models calculated with the Lead-DBS pipeline to a more elaborate software (OSS-DBS, also used to validate findings in the present article).
Segmentation of deep nuclei	Comparison to manual expert segmentations	[20]	This study led to the current default settings of Lead-DBS which were capable of segmenting STN and GPi nuclei almost as accurately as expert segmentations (comparison to interrater DICE scores and surface distances.
		[21]	External validation of the [20] study (above).
	MER/MUA	[9,22]	This study demonstrated high agreement between definitions of the subthalamic nucleus by microelectrode recordings and anatomical segmentations carried out by Lead-DBS.
Brain Shift Correction Algorithm	MER/MUA	[22]	This study compared the fit between microelectrode recordings before and after applying the brain shift correction implemented in Lead-DBS and showed significant increase in fit.
Sweetspot mapping	Synthetic Ground truth Datasets	[23]	This study compared various published concepts to carry out sweetspot mapping using a synthetic ground truth dataset. All concepts were implemented into Lead-DBS and the winning concept was chosen as the default parameter.
	Clinical Datasets	[24]	This study compared various proposed concepts of sweetspot mapping as implemented in Lead-DBS and their utility to estimate variance in out-of-sample data in an STN-DBS cohort of patients with PD (N=95).

Validated Concept	Validated using…	Study	Notes
Estimates of Behavioural Outcome in unseen data	Behavioral tasks	[25]	This study used Lead-DBS derived models to explain variance in changes of reaction time and movement velocity in a behavioral task setting as a function of STN-DBS in 20 patients with PD. ~56-76% of variance in behavioral changes explained (out of sample data).
		[26]	This study used Lead-DBS derived models to explain variance in motor learning in a behavioral task setting as a function of STN-DBS in 20 patients with PD. 33% of variance in behavioral changes explained (out of sample data).
Estimates of Clinical Outcome in unseen data	Clinical scores	[27]	This study showed a significant correlation between clinical improvements (%-UPDRS-III) in PD-patients undergoing STN-DBS with i) electrode placement (distance to an optimal target coordinate), ii) VTA coverage of the STN as modeled by Lead-DBS and iii) structural connectivity to the supplementary motor area seeding from the modeled VTA
		[28]	This study estimated clinical improvements (% UPDRS-III in STN-DBS for PD) based on structural and functional connectivity seeding from the modeled VTA. An optimal connectivity profile was calculated on a first cohort and used to estimate outcomes in an unseen second cohort operated by a second surgeon at a different center.
		[24]	This study demonstrated validity of predictive models on local, tract- and network-levels using Lead-DBS to estimate motor response in Parkinson's Disease (%-UPDRS-III).
		[29]	This study estimated clinical improvements (% TRS in VIM-DBS for ET) based on structural and functional connectivity seeding from the modeled VTA. The optimal connectivity profiles were used to estimate outcomes in unseen patients using a leave-one-out design. ~13-16% of variance in clinical improvements explained (out of sample data).

Validated Concept	Validated using…	Study	Notes
		[30]	This study estimated clinical side-effects (% BDI in STN-DBS for PD) based on structural connectivity seeding from the modeled VTA. A connectivity profile was associated with postoperative depression based on a first cohort and used to estimate outcomes in an unseen second cohort operated by a second surgeon at a different center (and vice-versa). A third test-cohort was used to further validate the model. ~10-33% of variance in clinical improvements explained (out of sample data).
		[31,32]	These studies established a tract that, when stimulated, would lead to improvements of OCD symptoms following DBS to either the ALIC or STN target. Results were cross-validated across targets, cohorts and centers.<br>~25-56% of variance in clinical improvements explained (out of sample data).
		[33]	External validation of the [32] study (above).
		[34]	External validation of the [32] study (above).
		[36]	External validation of the [32] study (above).
		[37]	External validation of the [32] study (above).
		[38]	Blinded validation of the [32] study (above).
		[39]	This study used functional connectivity as input for a machine learning model and showed that the connectome-based model was able to estimate STN-DBS outcome in 50 patients with Parkinson's Disease.

Validated Concept	Validated using…	Study	Notes
		40	This study showed that 6 neuroanatomical parameters computed by Lead-DBS (distance of each contact to the STN and the motor part of the STN, volume of the overlapping areas of the VTA and STN/ and motor part of the STN, and the number and ratio of fiber tracts through both the VTA and motor areas) were individually relevant to determine group differences between clinical optimal and nonoptimal outcomes. Additionally, the combined use of all 6 parameters suggested optimal contact selections with an accuracy of 73%.
		41	This study showed the relationship between the position of bilateral STN-DBS location of active contacts and clinical efficacy of the therapy on motor symptoms in 57 Parkinson's disease patients.
		42	This multicenter international study demonstrated the correlation between non-motor outcomes and DBS electrode location in 91 Parkinson's Disease patients.
Automated DBS programming	Clinical scores		This prospective randomized double-blinded study demonstrated that an automated data-driven algorithm that was based on Lead-DBS localizations (StimFit) could suggest stimulation parameters that led to non-inferior motor symptom improvements when compared to standard of care treatment in PD patients implanted in the STN.

Supplementary Methods

S3. Creation and Validation of the DBS Tractography Atlas Version 2 Tract References

Table S2. List of abbreviations used in Section S3.

**ac**	Anterior commissure
**atr**	Anterior thalamic radiation
**AQ**	Aquectuctus mesencephali
**A6m**	Medial subdivision of area 6
**A6ld**	Laterodorsal subdivision of area 6
**BA**	Brodman area
**Ca**	Caudate
**CB**	Cerebellum
**CC**	Corpus callosum
**CO**	Chiasma opticum
**CP**	Cerebral peduncle
**CQ**	Corpora quadrigemina
**crt**	Cerebello-rubro thalamic tract
**DN**	Dentate nucleus
**fnx**	Fornix
**fr**	Fasciculus retroflexus of Meynert
**FV**	Fourth ventricle
**GPe**	Globus pallidus externus
**GPi**	Globus pallidus internus
**LV**	Lateral ventricle
**MB**	Mammillary body
**ml**	Medial lemniscus
**mlf**	Medial longitudinal fasciculus
**mmt**	Mammillothalamic tract
**MO**	Medulla oblongata
**M1c**	Primary motor cortex (area M1, area 4)
**OCN**	Oculomotor nerve
**Ol**	Olive
**ON**	Optical nerve
**opt**	Optical tract
**PPN**	Pedunculopontine nucleus
**Pu**	Putamen
**RN**	Red nucleus
**rst**	Rubrospinal tract
**SCP**	Superior cerebellar peduncle

**SN**	Substantia nigra
**SP**	Septum pellucidum
**ST**	Stria terminalis
**STN**	Subthalamic nucleus
**Th**	Thalamus
**xSCP**	Decussation of superior cerebellar peduncle
**ZI**	Zona incerta

Table S3: V2 DBS Tractography Atlas tract references. Abbreviations: Ac = Nucleus accumbens, BA = Brodman area, DN = Dentate nucleus, Drtt = Dentatorubrothalamic tract, GPi = Globus pallidus internus, GPe = Globus pallidus externus, M1 = Primary motor cortex, MFG = Middle frontal gyrus, OC = Olfactory cortex, OFC = Orbitofrontal cortex, PC = Prefrontal cortex, PPN = Pedunculopontine nucleus, SFG = Superior frontal gyrus, SMA = Supplementary motor area, SN = Substantia nigra, STN = Subthalamic nucleus, Th = Thalamus, VIM = Ventral intermedius nucleus, VO = Ventralis oralis nucleus, VTA = Ventral tegmental area, vtaPP = Projection pathway from the ventral tegmental area.

Identification code	Complete anatomical name	Filename	Defined by	Based on	ROI
ID-01	Ansa lenticularis	al	[44]	Expert neuroanatomist's definition (EM), foundation: Morel atlas ([45,46])& CIT168 ([47])	see [44]
ID-02	Fasciculus lenticularis	lenf	[44]	Expert neuroanatomist's definition (EM), foundation: Morel atlas ([45,46]) & CIT168 ([47])	see [44]
ID-03	Anterior thalamic radiation	atr	[48]	HCP 1065	Th–PC

ID-04	Dentatorubrothalamic Tract	drtt-SMA	[48]	HCP 1065	DN–RN– VO – SMA (BA6)
ID-05	Dentatorubrothalamic Tract	drtt-M1	present study	HCP 1065	DN–RN – VIM – M1 (BA4)
ID-06	Non-decussating Dentatorubrothalamic Tract	ndrtt	[48]	HCP 1065	DN–RN – VIM – M1 (BA4)
ID-07	Medial Forebrain Bundle	mfb	[48]	HCP 1065	VTA–Ac–OC
ID-08	Subthalamic nucleus to globus pallidus internus connection (sensorimotor fibres)	STN-GPi-sensorimotor	[44]	Expert neuroanatomist's definition (EM), foundation: Morel atlas ([45,46]) & CIT168 ([47])	see [44]
ID-09	Subthalamic nucleus-to globus pallidus internus connection (associative fibres)	STN-GPi-associative	[44]	Expert neuroanatomist's definition (EM), foundation: Morel atlas ([45,46]) & CIT168 ([47])	see [44]
ID-10	Subthalamic nucleus to globus pallidus externus connection (sensorimotor fibres)	STN-GPe-sensorimotor	[44]	Expert neuroanatomist's definition (EM), foundation: Morel atlas	see [44]

				([45,46]) & CIT168 ([47])	
ID-11	Subthalamic nucleus-to globus pallidus externus connection (associative fibres)	STN-GPe-associative	[44]	Expert neuroanatomist's definition (EM), foundation: Morel atlas ([45,46]) & CIT168 ([47])	see [44]
ID-12	Globus pallidus externus to subthalamic nucleus connection (sensorimotor fibres)	GPe-STN-sensorimotor	[44]	Expert neuroanatomist's definition (EM), foundation: Morel atlas ([45,46]) & CIT168 ([47])	see [44]
ID-13	Globus pallidus externus to subthalamic nucleus connection (associative fibres)	GPe-STN-associative	[44]	Expert neuroanatomist's definition (EM), foundation: Morel atlas ([45,46]) & CIT168 ([47])	see [44]
ID-14	Subthalamic nucleus to substantia nigra connection	STN-SN	[49]	HCP 1065	STN–SN
ID-15	VTA projection pathway	VTApp	[48]	HCP 1065	DN–VTA–SFG–MFG–lateral OFC
ID-16	Globus pallidus internus to pedunculopontine nucleus connection	GPi-PPN	present study	HCP 1065	GPi–PPN (PPN segmentation according to[50])

ID-17	Pedunculopontine nucleus to globus pallidus connection	PPN-GP	present study	HCP 1065	Manual ROI definitions
ID-18	Pedunculopontine nucleus to M1 connection	PPN-M1	present study	HCP 1065	PPN–M1 (BA4)
ID-19	Pedunculopontine nucleus to SMA connection	PPN-SMA	present study	HCP 1065	PPN–SMA (BA6)
ID-20	Subthalamic nucleus to pedunculopontine nucleus connection	STN-PPN	present study	HCP 1065	STN-PPN (PPN segmentation according to[50])
ID-21	Corticospinal tract	cst	present study	HCP 1065	Motor & Premotor regions defined by HMAT atlas[51] – manual midbrain slice
ID-22	Medial Lemniscus	ml	Present study	HCP 1065	Manual ROI definitions
ID-23	Rubrocortical tract	rcot	present study	HCP 1065	Manual ROI definitions
ID-24	Rubrospinal tract	rst	present study	HCP 1065	Manual ROI definitions
ID-25	Rubrocerebellar tract	rcet	present study	HCP 1065	Manual ROI definitions
ID-26	Rubroolivary tract	rot	present study	HCP 1065	Manual ROI definitions

ID-27	STN to Brodman area 1 & 2 & 3 connection	STN-BA1BA2BA3	present study	Cortical Atlas by [52] & HCP 1065	STN – Somatosensory Cortex (1° , 2°, 3°)
ID-28	STN to Brodman area 4 connection	STN-BA4	present study	Cortical Atlas by & HCP 1065	STN – M1
ID-29	STN to Brodman area 6 connection	STN-BA6	present study	Cortical Atlas by [52] & HCP 1065	STN – SMA
ID-30	STN to Brodman area 8 connection	STN-BA8	present study	Cortical Atlas by [52] & HCP 1065	STN – frontal eye fields
ID-31	STN to Brodman area 10 connection	STN-BA10	present study	Cortical Atlas by [52] & HCP 1065	STN – Fronto-Parietal cortex
ID-32	STN to Brodman area 13 connection	STN-BA13	present study	Cortical Atlas by [52] & HCP 1065	STN – Insular cortex
ID-33	STN to Brodman area 25 connection	STN-BA25	present study	Cortical Atlas by [52] & HCP 1065	STN – Subguneal anterior cingulate cortex
ID-34	STN to Brodman area 24 & 32 connection	STN-BA24BA32	present study	Cortical Atlas by [52] & HCP 1065	STN – Dorsal anterior Cingulate Cortex & Preguneal Anterior

					Cingulate Cortex
ID-35	STN to Brodman area 45 and 47 connection	STN-BA45BA47	present study	Cortical Atlas by [52]<br><br>& HCP 1065	STN – Inferior Frontal Gyrus

Comparative Anatomical Validations of Tract Findings

Multiple network mapping approaches have used data-driven connectomes derived from diffusion-weighted imaging based tractography[31,32,53,54]. However, especially in the subthalamic region, these datasets often lack accuracy regarding thin projection bundles, such as the pallidothalamic projections (ansa and fasciculus lenticulares), Edinger's comb fibers or the cerebellothalamic pathways[55–59]. To overcome this limitation, a key development was the concept of manually curated pathway atlases defined using prior anatomical knowledge[60,61]. These datasets comprise meticulously curated pathways and are hence free from false-positive connections (tracts that do not exist in the brain). However, one downside of these atlases is that tracts that the anatomical team has not defined cannot be identified as critical for DBS success by post-hoc use of the atlas. This could lead to false-negative conclusions (not seeing tracts that exist in the brain). For instance, certainly the most accurate atlas to date defined by Petersen et al., lacks bundles such as the ansa subthalamica[56], connections between STN and PPN or striatonigral connections, among others, simply because an anatomical team has to focus on a number of tracts and cannot exhaustively include all tracts that exist in the brain. Furthermore, the STN receives cortical (hyperdirect) input from the entire frontal cortex[62,63] , which are not exhaustively represented in typical pathway atlases. To overcome this limitation, here, we compiled existing atlases[55,56] comprising cortical and subcortical pathways traversing the subthalamus and added missing connections based on anatomical data (supplementary table S3). For instance, pallidosubthalamic and pallidothalamic projections were informed based on the Basal Ganglia Pathway Atlas [56], while most other connections were defined based on the DBS Tractography atlas [55]. Missing connections, not represented in any atlas were reconstructed following the exact same methodology used to create the latter atlas, as described in detail elsewhere[64]. Using a diffusion template[65] compiled from 1065 participants of the

Human Connectome Project[66], connections between manually defined seed regions were calculated using DSI Studio software using the generalized q-Sampling Imaging algorithm [67]. Seed regions were defined using various atlases (Table S3) and resulting tracts were meticulously validated by comparing them to cadaveric dissection studies, postmortem imaging, histological and text-book data (below). The tracts were critically examined in both their two-dimensional and three-dimensional configuration and compared with various macroscopic and microscopic data, findings from tracing-studies as well as anatomical illustrations and confirmed by expert anatomist and neuroimaging co-authors (HF, GPS, SK, AK, CK, GS, VM, EM). The Tracts were visualized with the open-source software 3D-Slicer (https://www.slicer.org[68]) Version 5.2.2. A 100-micron 7T MRI ex vivo scan, normalized into the MNI ICBM 2009b NLIN ASYM template[69,70], served as the template for the illustrations. Please note that the colors used to illustrate the tracts were chosen arbitrarily and do not refer to the colour scheme applied in the paper. If not explicitly specified, the specimens depicted are of human origin. As a result, this revised version 2 of the DBS tractography atlas[55] consists of a more exhaustive set of connections from and to the STN.

IDs-01 & 02: Ansa lenticularis, Fasciculus lenticularis

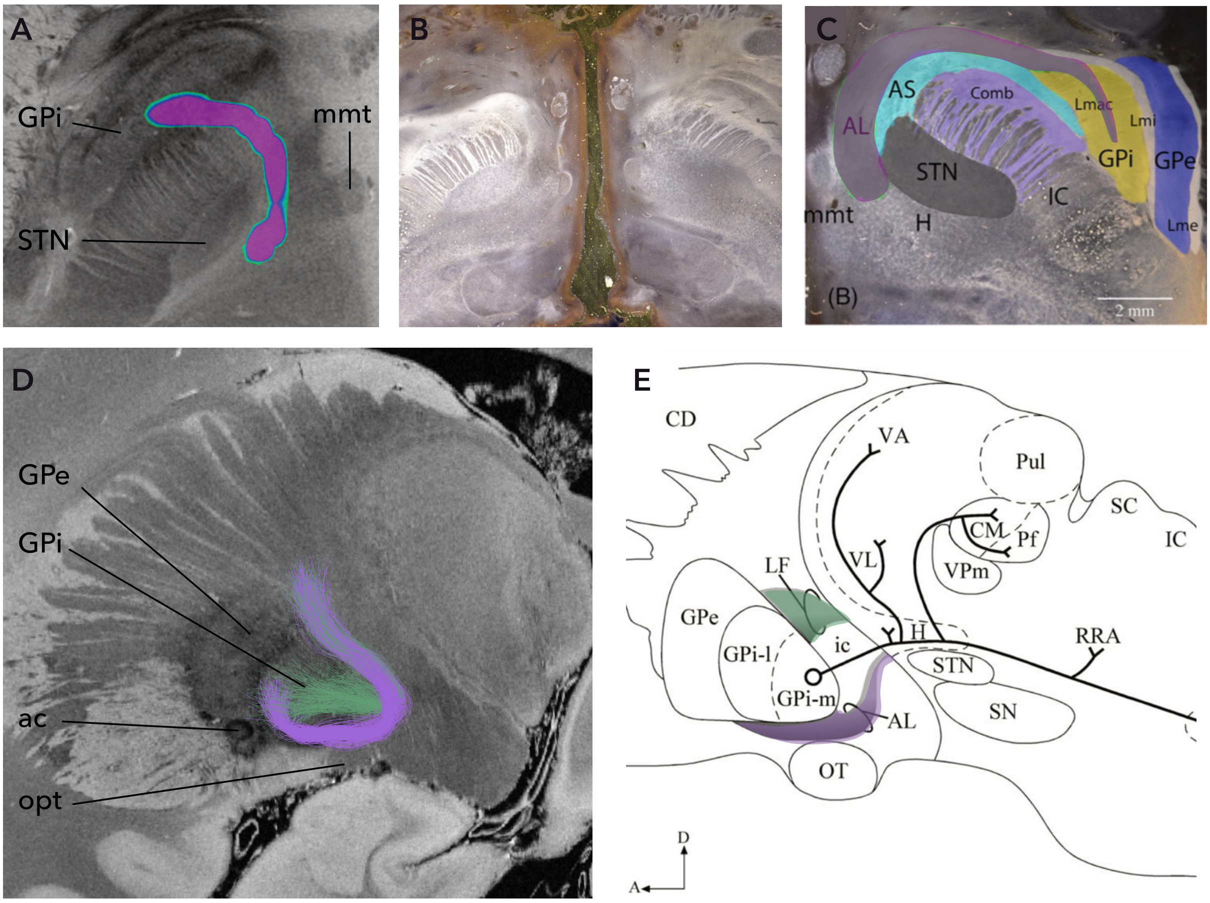

Figure S2. A Axial section, view from superior. Ansa lenticularis (al) shown in purple. **B** Dark field microscopy of the same sectional plane according to [71]. Adapted with permission from the authors. **C** Corresponding anatomical definitions adapted from [72]. Illustrations are used with permission of John Wiley & Sons – Books from [72] permission conveyed through Copyright Clearance Center, Inc. The license restrictions of the original publication apply to modification & reuse. **D** 3D-visualization of the al (purple) and fasciculus lenticularis (fl; green). Sagittal plane, right hemisphere. **E** Sagittal anatomical illustration according to [73]. Illustrations for E are reprinted from [73] with permission from Elsevier. The license restrictions of the original publication apply to modification & reuse.

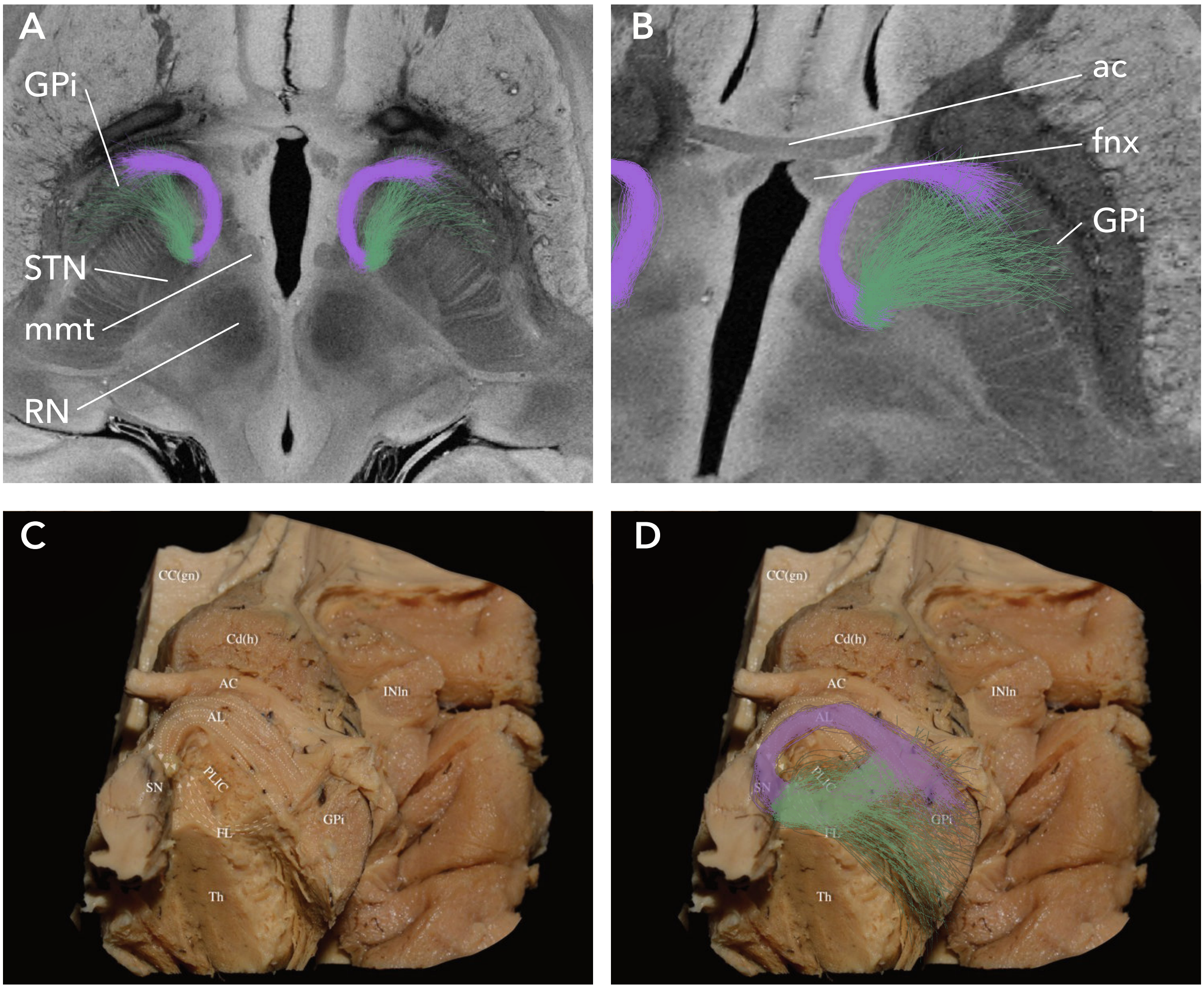

Figure S3. View from inferior to the al in purple and the fl in green. **A** Sectional plane more posterior, and **B** more anterior for enhanced spatial understanding. **C** Oblique-inferior view of human white matter fibres preparation kindly provided by Georgios Skandalakis. **D** Specimen with superimposed V2-tracts.

ID-03: Anterior thalamic radiation

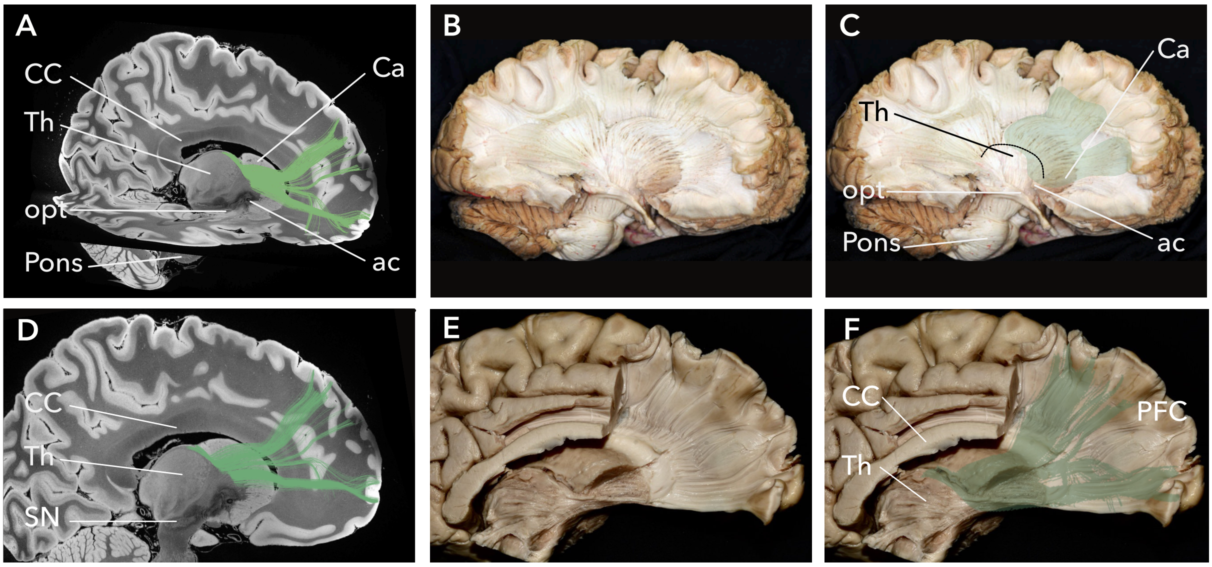

Figure S4. **A** Right anterior thalamic radiation (atr) displayed in green in a parasagittal section. Axial plane overlay added for context. **B** Fibre dissection of the atr, kindly provided by Vanessa Milanese Holanda. **C** Fibres of atr highlighted in green. **D** Left atr shown in a parasagittal section. **E** White matter preparation of the atr, kindly provided by Georgios Skandalakis. **F** Superimposed visualization of the left atr.

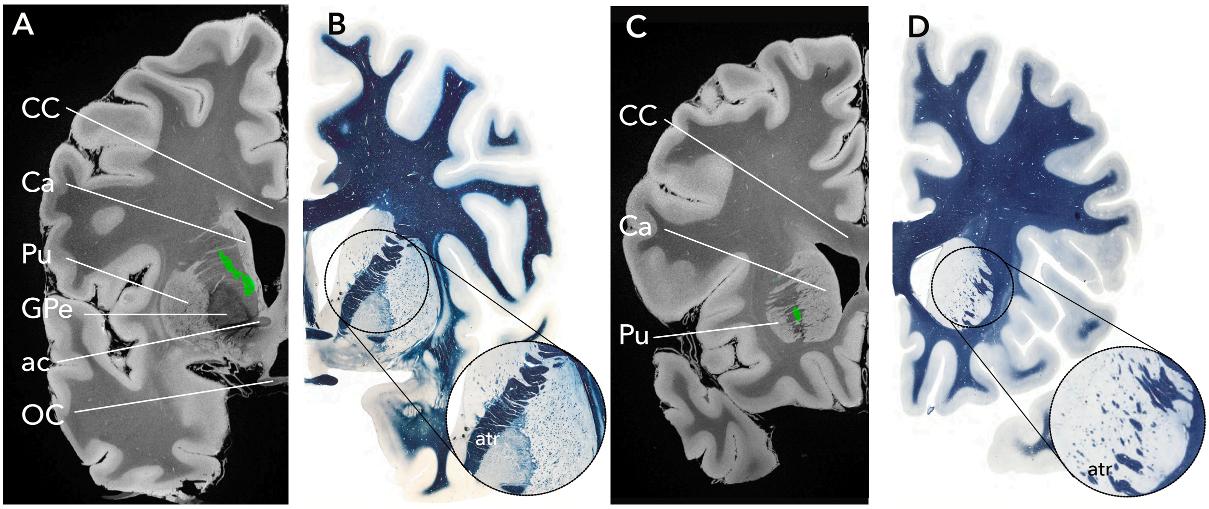

Figure S5. Comparative analysis of the atr in coronal sections. **A** Coronal section, anterior view. Atr is shown in green. **B** Respective histological section, indicating that our atr configuration is similar. **C-D** This also applies in more anterior coronal sections. B & D Illustration adapted with permission from Mai JK & Majtanik M (2015), Atlas of the Human Brain 4th edition, Elsevier Academic Press [74]. The license restrictions of the original publication apply to modification & reuse.

IDs-04-06: Dentatorubrothalamic tract (04 & 05), Non-decussating dentatorubrothalamic tract (06)

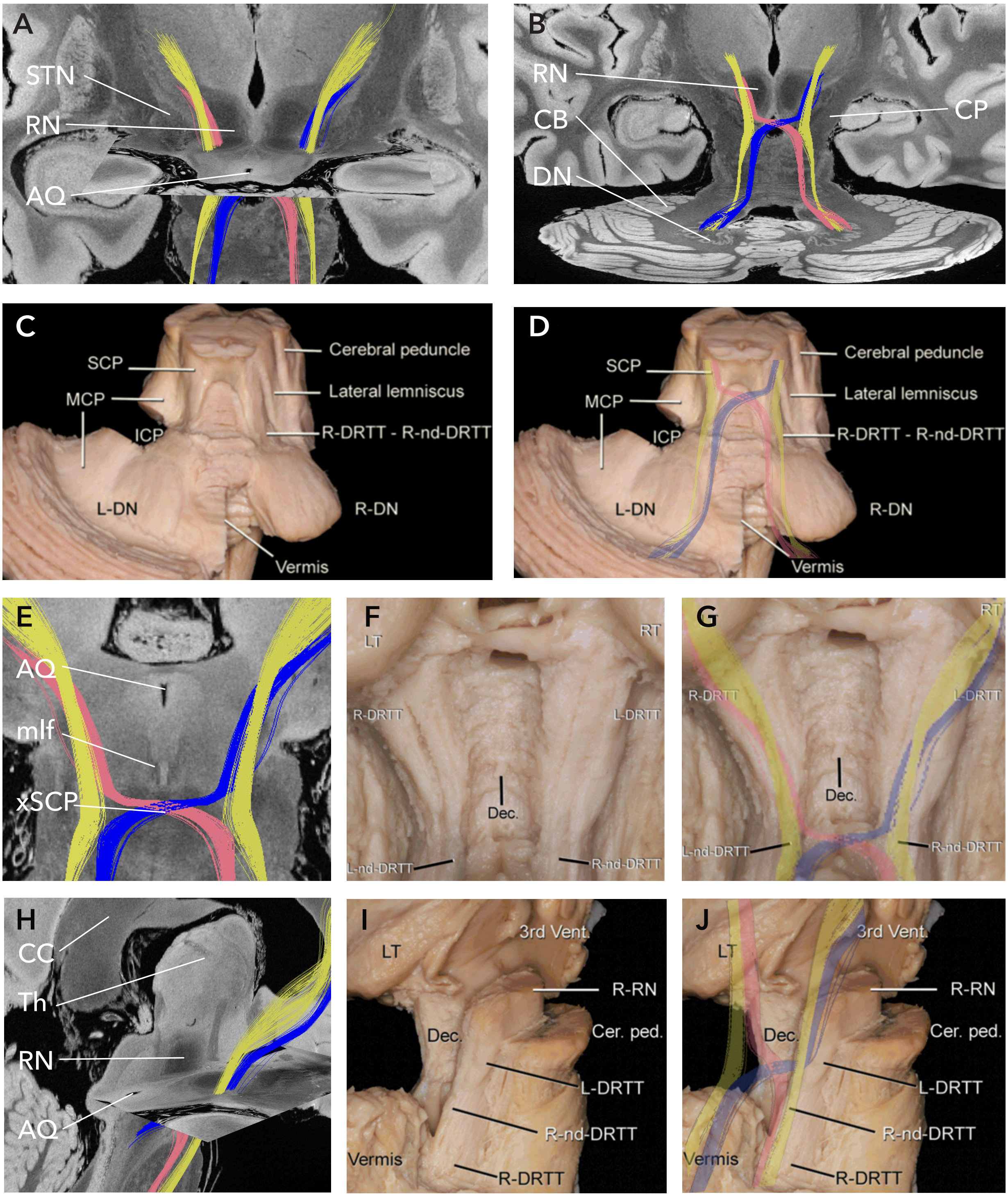

Figure S6. A Coronal Section, posterior view with additional axial section at the level of the red nucleus (RN; A) and **B** dentate nucleus. Yellow: non-decussating dentatorubrothalamic tract (ndrtt), red: right dentatorubrothalamic tract (drtt), blue: left drtt. **C** Fibre dissection by [75] **D** with superimposed tracts. **E** Posterior

coronal section **F** Corresponding fibre dissection [75] **G** with overlaid tracts. **H** Parasagittal section, right oblique view with axial section through the RN. **I** Fibre dissection [75] at comparable level **J** correlated with our tracts. Illustrations for C-D, F-G, I-J are adapted with permission from Meola et al (2016) [75]. The non-decussating pathway of the dentatorubrothalamic tract in humans: Human connectome-based tractographic study and microdissection validation. Journal of Neurosurger, 2016. The license restrictions of the original publication apply to modification & reuse.

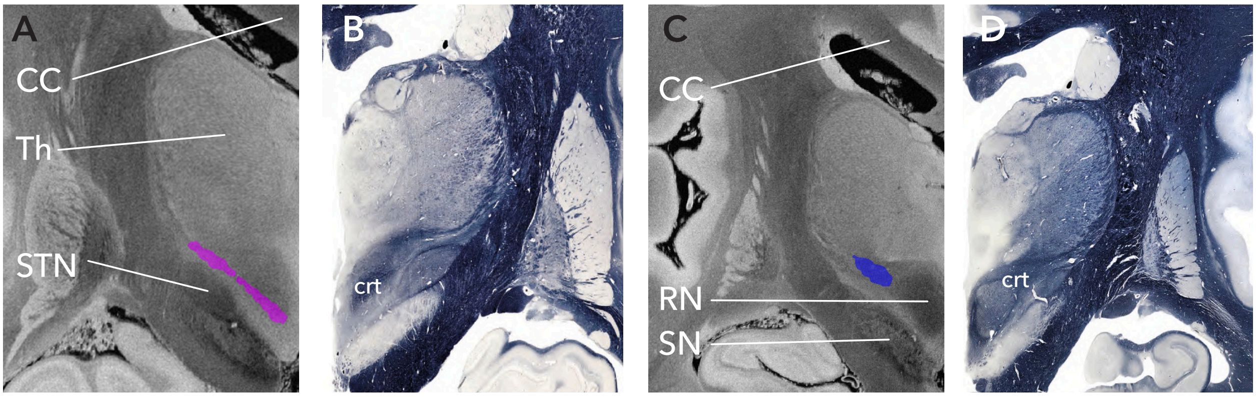

Figure S7. **A** Anterior coronal section, drtt in magenta (Version 1, see [48]) with **B** corresponding histological section by [74]. **C** drtt in blue (Version 2) was added to describe the anatomical extension of the tract in even finer detail. **D** Corresponding Weigert fibre stain illustrated by [74]. Illustration adapted with permission from Mai JK & Majtanik M (2015), Atlas of the Human Brain 4th edition, Elsevier Academic Press. The license restrictions of the original publication apply to modification & reuse.

ID-07: Medial forebrain bundle

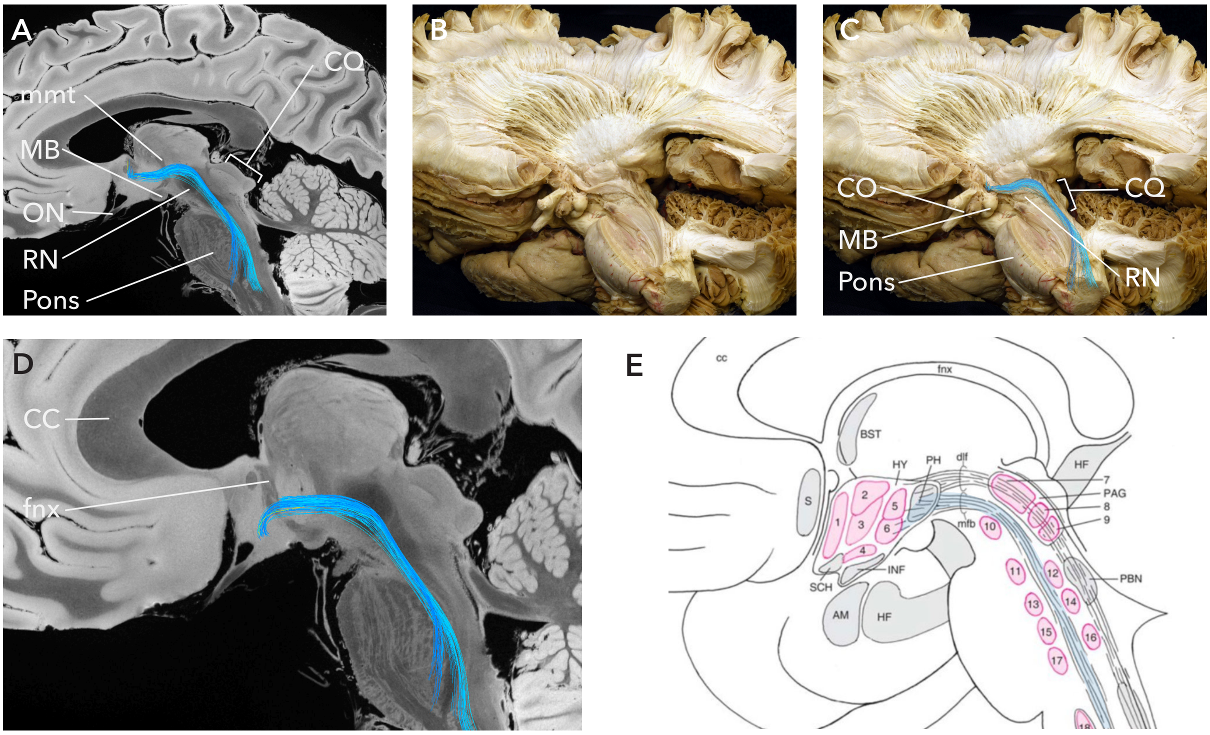

Figure S8. A Sagittal plane, right hemisphere. Right medial forebrain bundle (mfb) shown in turquoise. **B** Fibre dissection of the mfb, kindly contributed by Vanessa Milanese Holanda. **C** Superimposition of our mfb, demonstrating a course comparable to that in B. **D** Sagittal section, visualization of the right mfb. **E** Dimensions and course comparable to the mfb. Illustrated adapted from Nieuwenhuys et al, 2008, The Human Central Nervous System, Springer Berlin, Heidelberg. Reproduced with permission from SNCSC[76]. The license restrictions of the original publication apply to modification & reuse. Blue color was added for emphasis. Critically, this tract represents the anatomical definition of the medial forebrain bundle and should not be confused with the tract defined along the surgical target with a similar name (termed ‘superolateral MFB’ [77]), which has been included here due to its clinical importance and referred to as VTA projection pathway (VTApp, ID-15), following the renaming suggested by Coenen et al. [77]. While the mfb proper (anatomical textbook definition [78]) follows a transhypothalamic route (as shown here and also confirmed by Coenen et al. [77]), the tract associated with the sl-MFB/VTApp surgical target represents a subsection of the internal capsule [79].

IDs-08 & 09: Subthalamic nucleus – Globus pallidus internus (sensorimotor), Subthalamic nucleus – Globus pallidus internus (associative)

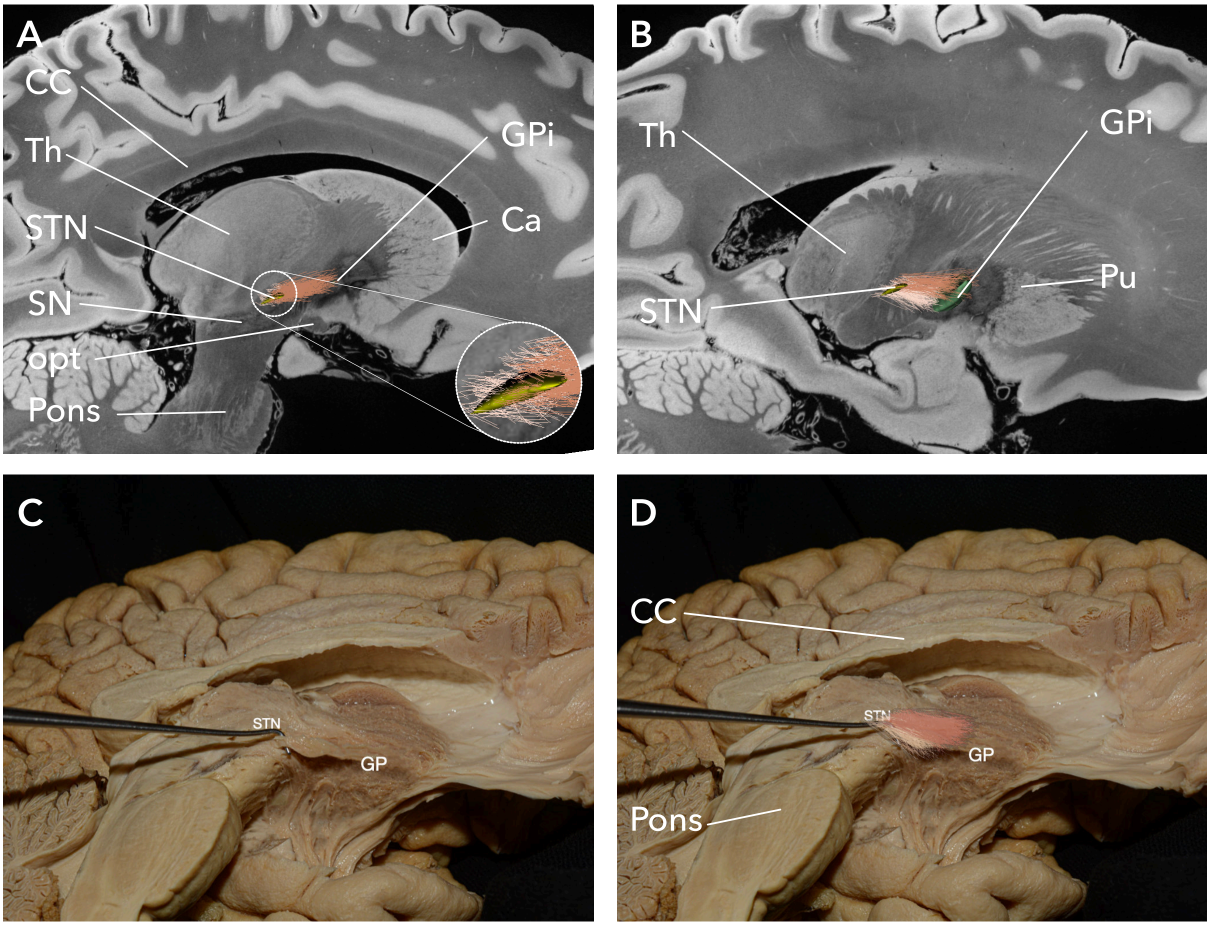

Figure S9. A View from right to left hemisphere. STN-GPi connections (associative fibre tracts) shown in pink. STN-GPi connections (sensorimotor fibre tracts) displayed in light pink. **B** Sectional plane selected further left-laterally to be able to show deeper fibre tracts. A & B STN 3D-rendered in yellow, GPi in green; both structures derived from the DISTAL-Atlas [80]. **C** Human brain cadaver, kindly provided by Georgios Skandalakis, after removal of the right hemisphere. View of left hemisphere from right. Fibre connections between STN & GP were dissected using the Klingler method. Orientation of fibre tracts in C comparable to that of our tracts, see **D**. Superimposition of STN-GPi connections. Please note that further differentiation between associative/ sensorimotor connections as well as directions cannot be distinguished by the present fibre dissection. For more information on anatomical justification of those tracts please refer to the original work by [44].

IDs-10-13: Subthalamic nucleus – Globus pallidus externus (sensorimotor), Subthalamic nucleus – Globus pallidus externus (associative), Globus pallidus externus - Subthalamic nucleus (sensorimotor), Globus pallidus externus - Subthalamic nucleus (associative)

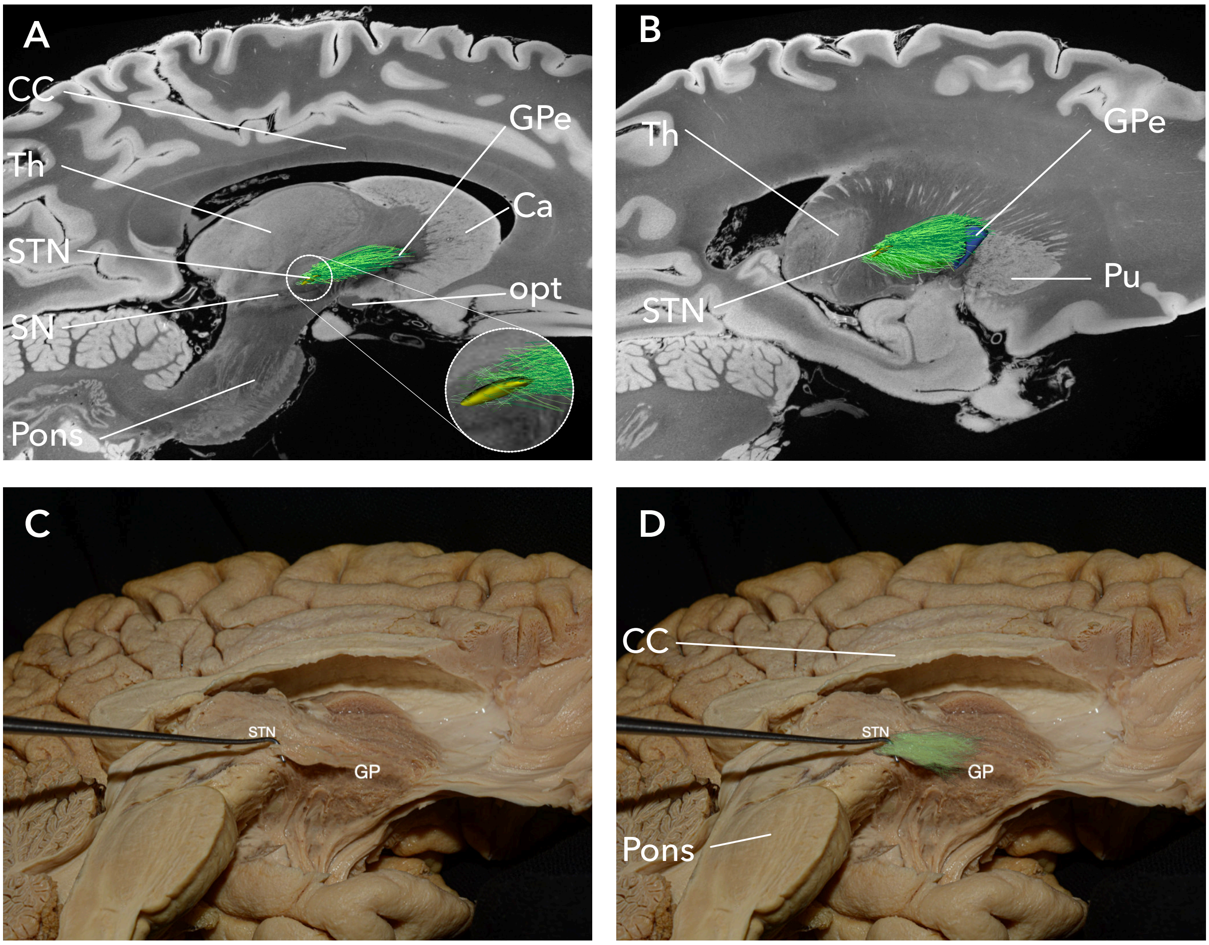

Figure S10. A View from right to left hemisphere. GPe-STN connections (sensorimotor/ associative fibre tracts) shown in lighter green; STN-GPe connections (sensorimotor/ associative fibre tracts) displayed in darker green. **B** Sectional plane selected further left-laterally to be able to show deeper fibre tracts. A & B STN 3D-rendered in yellow, GPe in blue; both structures derived from the DISTAL-Atlas[80]. **C** Human brain cadaver, kindly contributed by Georgios Skandalakis, after removal of the right hemisphere. Medial view of left hemisphere showing the connections between STN & GP prepared using the Klingler method. **D** Configuration of fibre tracts in C comparable to that of our tracts. Please note that further differentiation between associative/ sensorimotor connections as well as directions cannot be shown in the present fibre dissection. For more information on anatomical justification of those tracts please refer to the original work by [44].

ID-14: Subthalamic nucleus – Substantia nigra

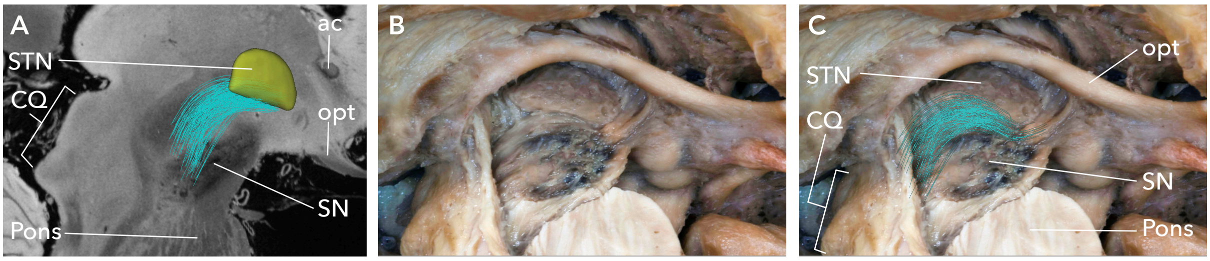

Figure S11. A Parasagittal section, right view. Right STN- Substantia nigra (SN) shown in turquoise. STN visualized in yellow (DISTAL Atlas[80]). **B** Fibre dissection of STN-SN-tracts kindly provided by Vanessa Milanese Holanda. Orientation of fibres seen in the specimen resemble those of V2-STN-SN-tract, see **C**.

ID-16: Globus pallidus internus – Pedunculopontine nucleus

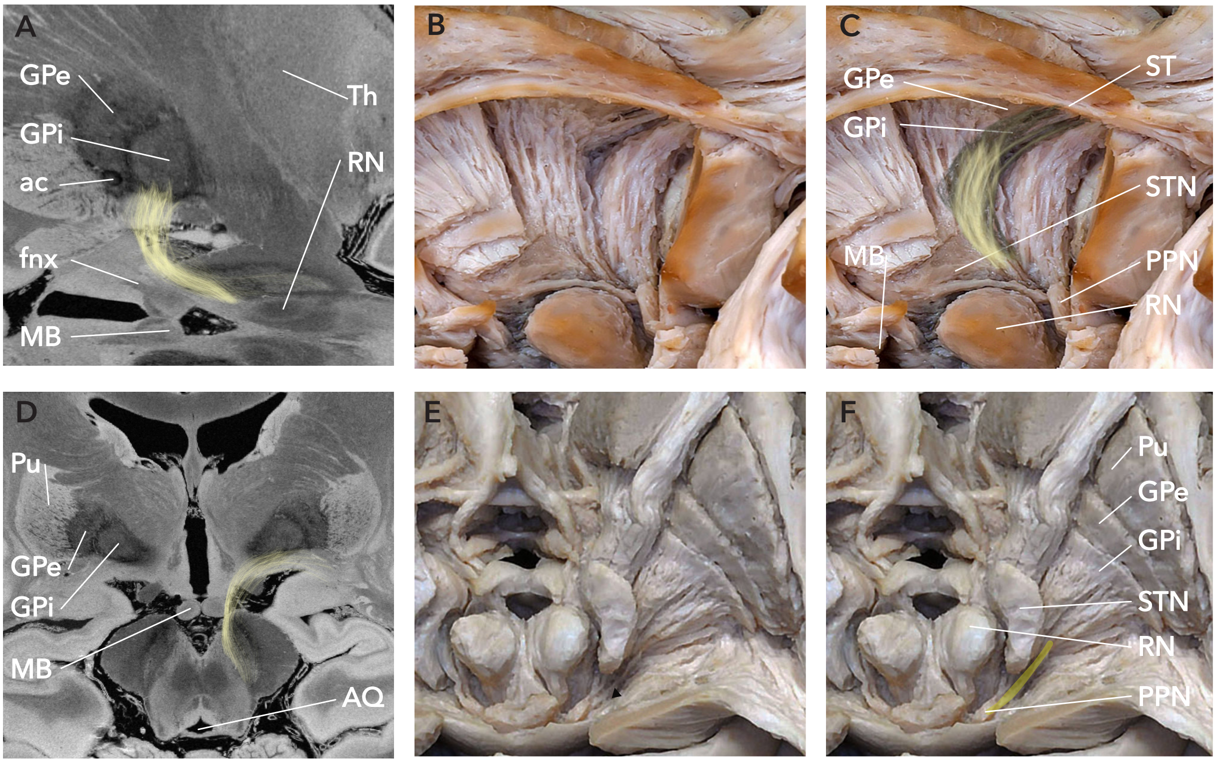

Figure S12. A Parasagittal section combined with axial section, view from the left. Right globus pallidus internus (GPi) to PPN connection shown in yellow. **B-C** Fibre dissection of this tract kindly provided by Vanessa Milanese Holanda. **C** Superimposed V2-tract implicates equivalent fibre orientation as seen in specimen. **D** Posterosuperior view. **E** Comparable section of white matter fibre dissection kindly contributed by Vanessa Milanese Holanda. **F** GPi-PPN-Tract highlighted in yellow.

ID-17: Pedunculopontine nucleus - Globus pallidus

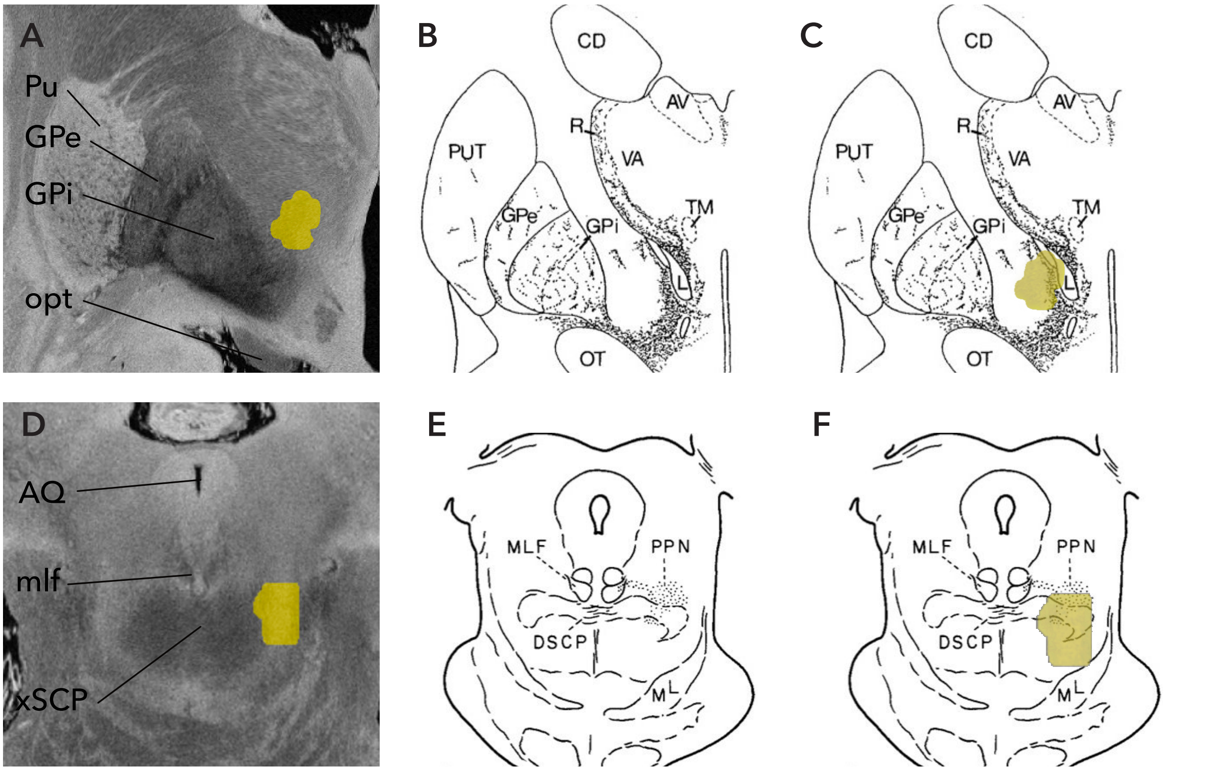

Figure S13. A Coronal section, anterior view. PPN-GPe-connection visualized in dark yellow. **B** [81] studied efferent projections of the PPN in squirrel monkeys using anterograde tracers. After injection to the central part of the PPN distribution of labeled neuronal profiles were summarized in the drawing shown in B. Tracings reached both GPi and GPe, even though the GPi was more densely innervated. **C** Our V2-tract has similar dimensions, and leads to more ventral portions of the GPe (not shown here) as similarly outlined in the study. **D** Posterior coronal section. **E** [82] described cells that were labelled after injection of the GP(i) in rhesus monkeys. Tracings could be followed to the PPN. **F** Our tract shows similar dimensions in the corresponding section. Illustrations for B-C are used with permission of John Wiley & Sons – Books from [81] . Permission conveyed through Copyright Clearance Center, Inc. Illustrations for E-F are used with permission of John Wiley & Sons – Books from [82] . Permission conveyed through Copyright Clearance Center, Inc. The license restrictions of the original publication apply to modification & reuse.

ID-18: Pedunculopontine nucleus –Primary motor area

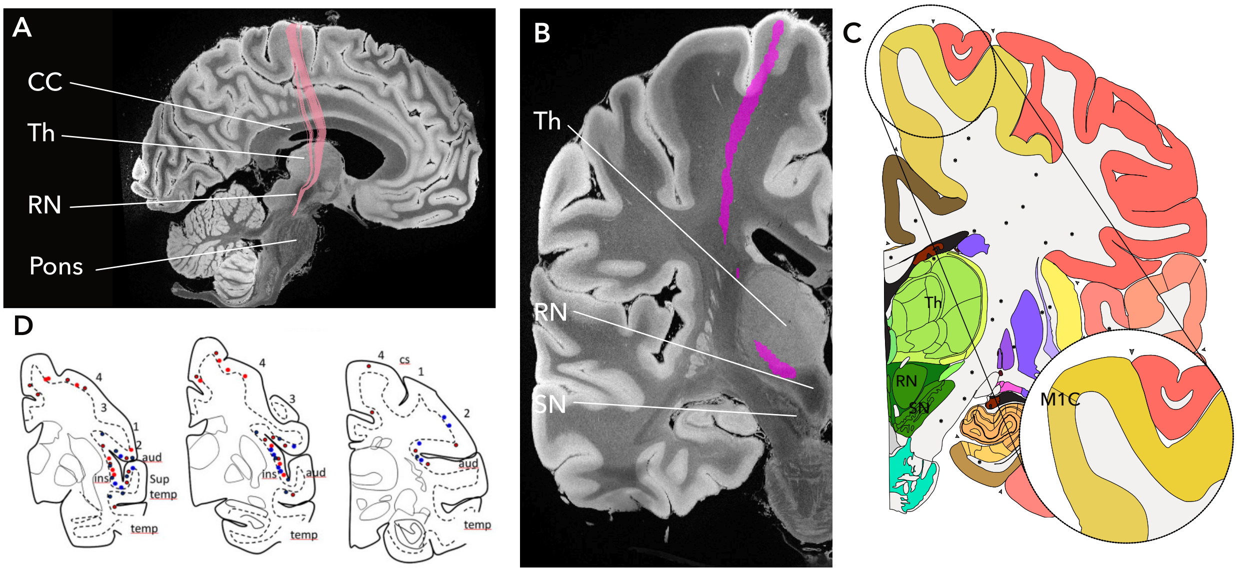

Figure S14. A Right parasagittal view, right PPN-M1-tract shown in red. **B** Coronal section, view from anterior. Tract reaches the primary motor area (M1; area 4) . See **C** corresponding section adapted from [83]. **D** captures average cortical projections to area 4 after injections to the PPN (red dots) in a monkey (Cercopithecus aethiops). Study by [84]. Illustrations for C adapted with permission from authors. Illustrations for D are reprinted Anatomical evidence for functional diversity in the mesencephalic locomotor region of primates. NeuroImage **147**, 66–78 (2017) with permission from Elsevier. The license restrictions of the original publication apply to modification & reuse.

ID-19: Pedunculopontine nucleus – Supplementary motor area

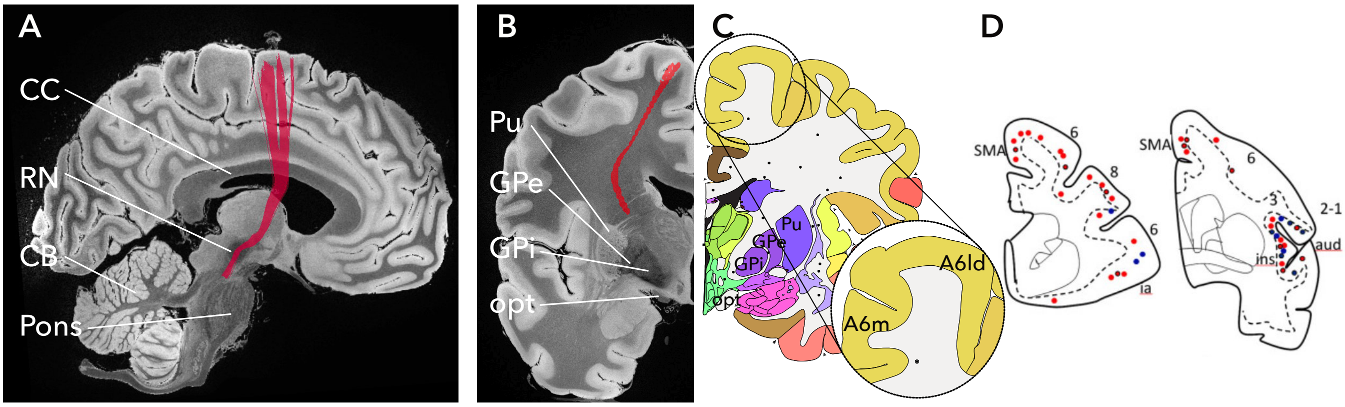

Figure S15. **A** Right parasagittal view, right PPN-SMA-tract shown in red. **B** Coronal section, view from anterior. Tract reaches the SMA (parts of area 6) . See **C** corresponding section adapted from [83]. **D** captures average cortical projections to area 6 after injections to the PPN (red dots) in a monkey (cercopithecus aethiops). Study by [84]. Illustrations for C adapted with permission from authors. Illustrations for D are reprinted Anatomical evidence for functional diversity in the mesencephalic locomotor region of primates. NeuroImage **147**, 66–78 (2017) with permission from Elsevier. The license restrictions of the original publication apply to modification & reuse.

ID-20: Subthalamic nucleus – Pedunculopontine nucleus

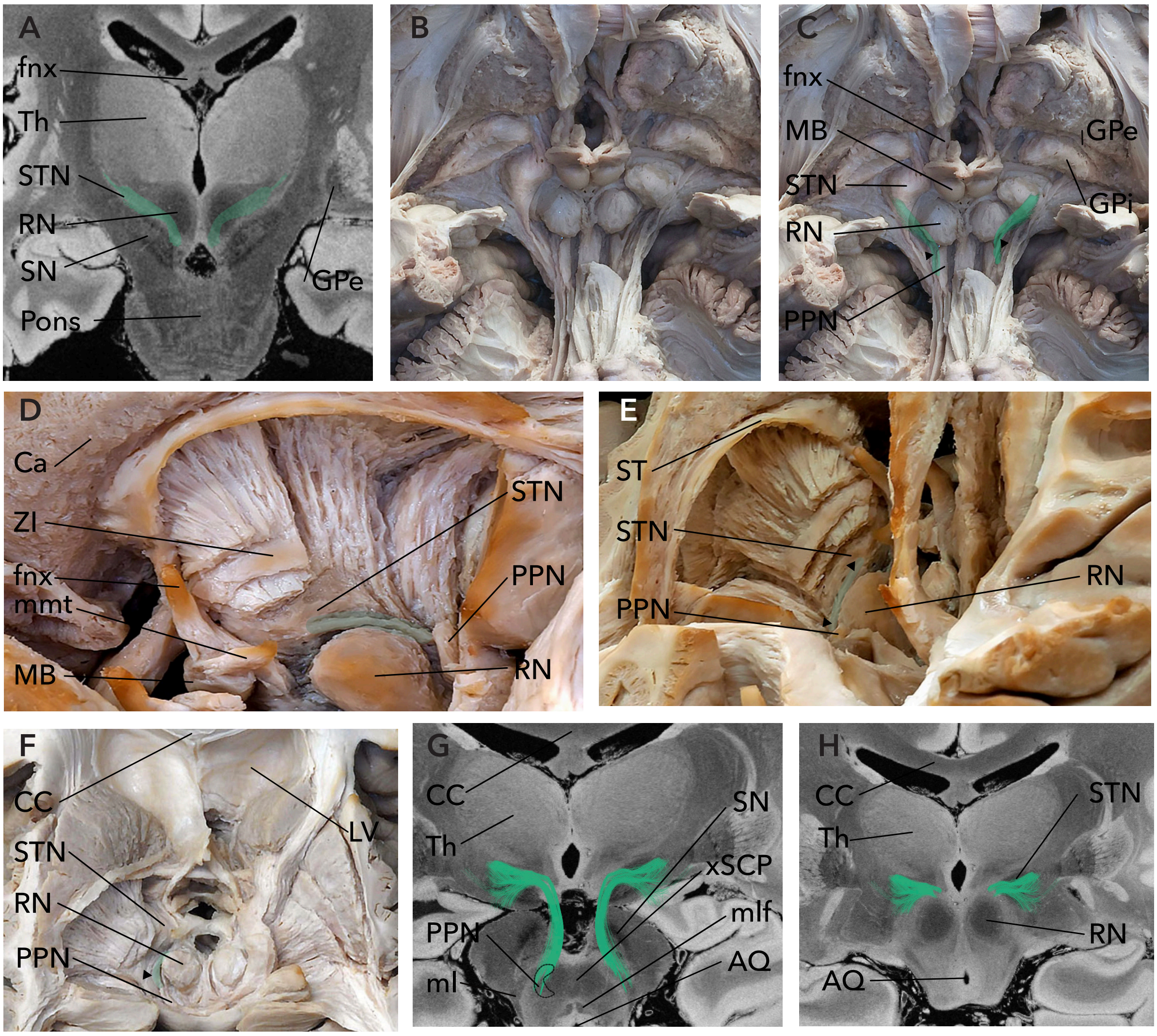

Figure S16. A Coronal section, anterior perspective. Subthalamic nucleus (STN) to pedunculopontine nucleus (PPN) connection shown in green. **B** Anterior view of white matter fibre dissection showing the connections between STN and PPN. **C** Superimposed STN-PPN V2-tracts. **D-E** Superior medial fibre dissections of the STN-PPN-tract. **F** Superior view at level of RN with STN-PPN tract highlighted in green. The V2-tract shows similar dimensions as demonstrated in **G-H** at different axial sectional planes for better illustration of its spatial configuration. Fibre dissections in B-F were kindly contributed by Vanessa Milanese Holanda.

ID-21: Corticospinal tract

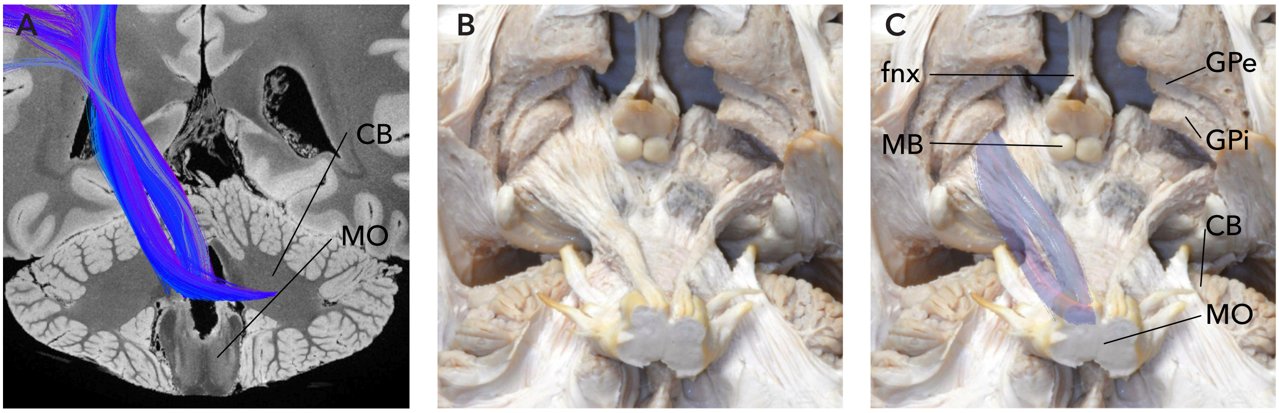

Figure S17. A Anterior view of the right corticospinal tract (cst). **B** Inferior-anterior view demonstrating the fibre dissection of the cst kindly provided by Vanessa Milanese Holanda. **C** Our tract shows similar alignment and dimensions in the corresponding section plane.

ID-22: Medial lemniscus

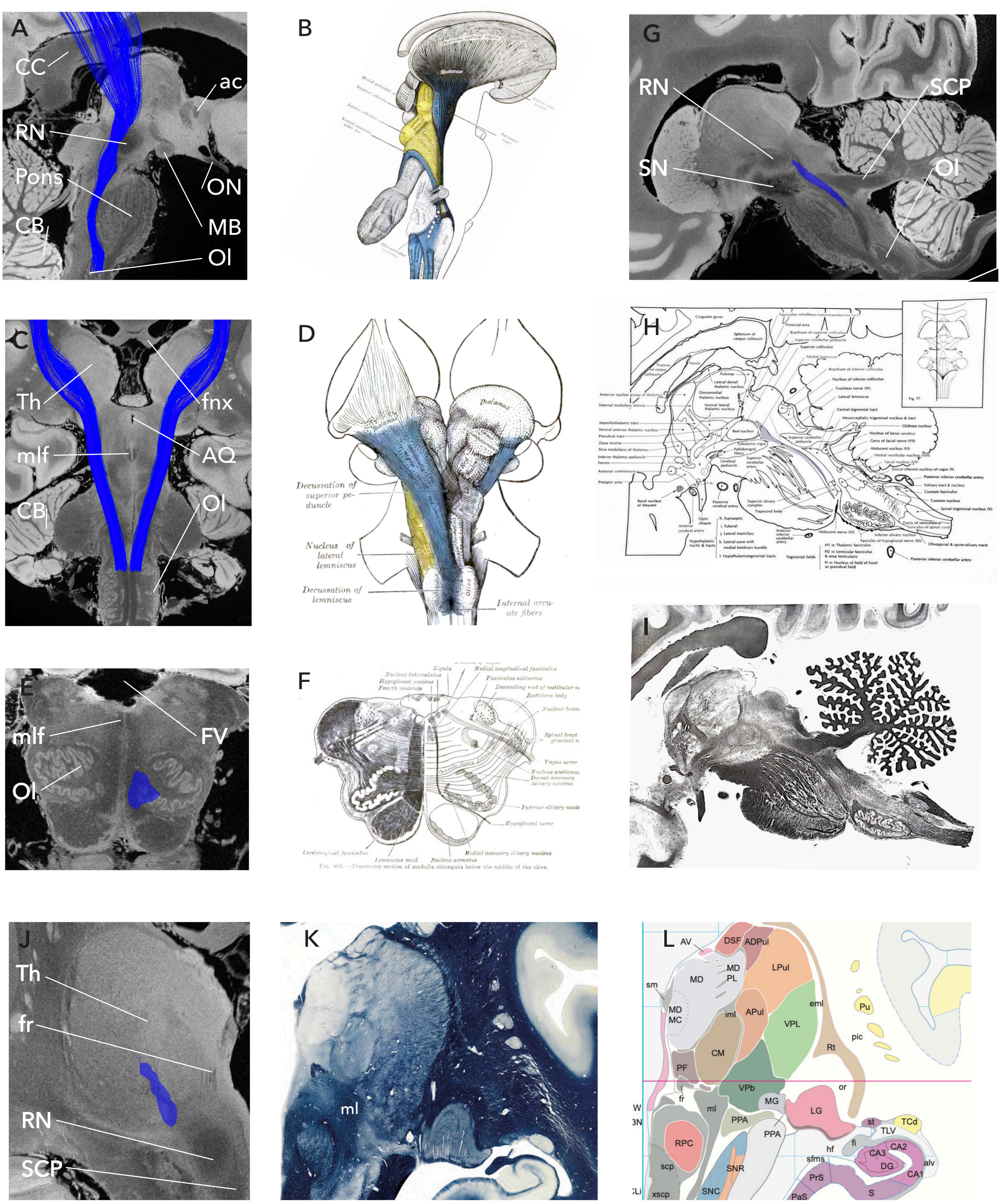

Figure S18. A Parasagittal section. The Medial Lemniscus (ml) depicted in blue. **B** Anatomical drawing by [85] of the ml in blue indicates comparable dimensions and orientation as our V2-tract. **C** Anterior coronal view of both the right and left ml and a **D** corresponding illustration by [85]. **E** Axial section through the medulla and corresponding anatomical scheme by [85] in **F** indicates that our ml shows equivalent spatial configuration. **G** Parasagittal section, view from left. Our ml presents the same location as indicated by [86] (illustration modified:

ml highlighted in blue) in **H-I** illustrates the corresponding microscopic section obtained from DeArmond et al, 1989, Structure of the Human Brain, a photographic atlas [86]. Reproduced with permission of the licensor through PLSclear. The license restrictions of the original publication apply to modification & reuse. **J** Coronal section through the red nucleus viewed from its anterior surface. Ml shown in blue overlaps with ml-position adapted from [74] see **K** associated Weigert fibre stain with **L** corresponding annotations. Illustration for K-L adapted with permission from Mai JK & Majtanik M (2015), Atlas of the Human Brain 4th edition, Elsevier Academic Press. The license restrictions of the original publication apply to modification & reuse.

IDs-21 & 22: Corticospinal tract, Medial lemniscus

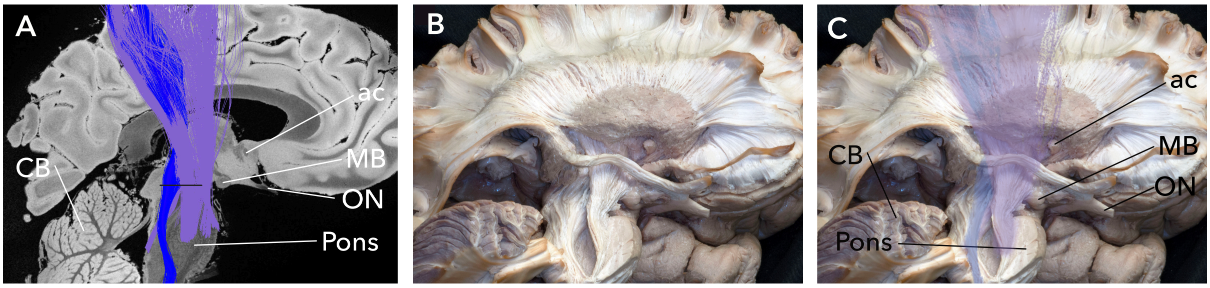

Figure S19. A Parasagittal section, right cst (purple) and ml (blue) viewed from the right. **B** Lateral view of white matter dissection kindly provided by Vanessa Milanese Holanda. **C** Spatial relation of our tracts similar to their equivalents demonstrated in B.

ID-23: Rubrocortical tract

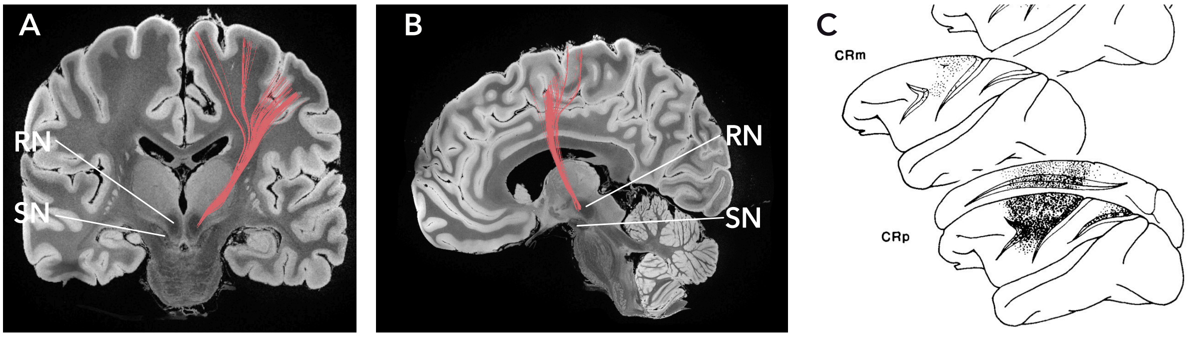

Figure S20. A Coronal section, anterior view of left rubro-cortical tract (rcot) displayed in red. **B** Parasagittal section. **C** [87] studied the cortical projections of the parvocellular (Crp) and magnocellular (Crm) divisions of the RN in monkeys (macaca mulatta and macaca fascicularis) with retrograde tracing methods. C shows a summary of the relative densities of those projections which pointed mostly to M1 and SMA . V2-tracts lead to same areas and synopsizes magnocellular and parvocellular projections. Illustrations for C are used with permission of John

Wiley & Sons – Books from [87] permission conveyed through Copyright Clearance Center, Inc. The license restrictions of the original publication apply to modification & reuse.

ID-24: Rubrospinal tract

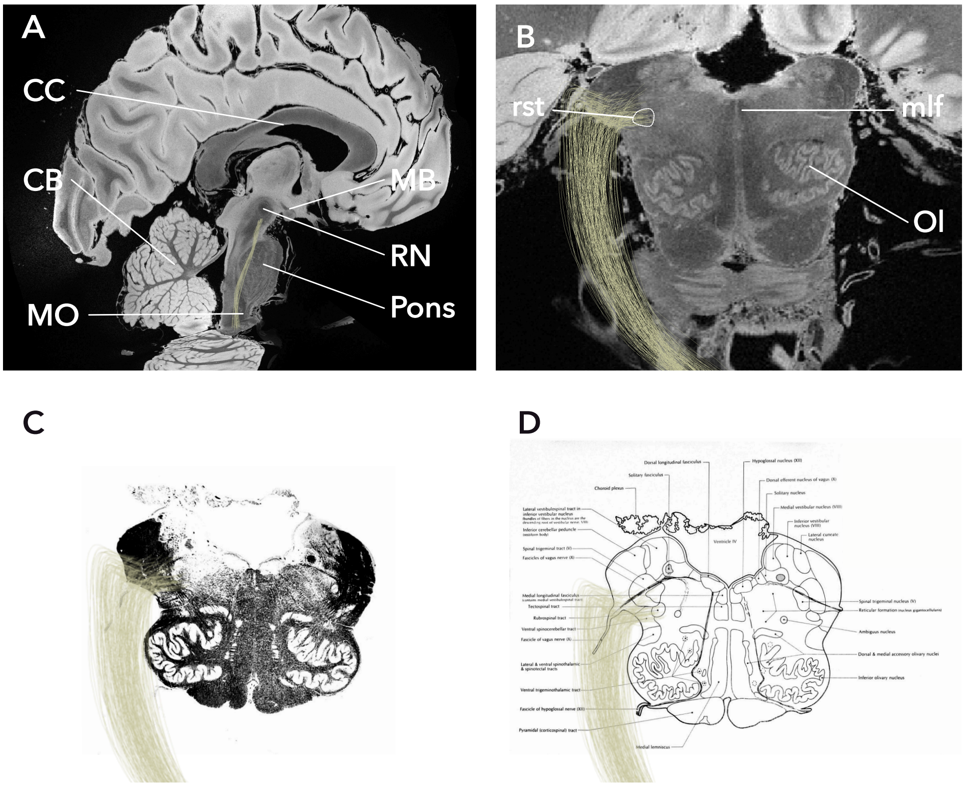

Figure S21. A Parasagittal section with axial section through medulla. Oblique view from right. Rubro-spinal tract (rst) shown in yellow. **B** Axial section. Superior view. **C** Microscopic picture of the same section **D** with corresponding annotations. The V2-tract converge at the site, which is expected to contain rubro-spinal fibres specifically. C-D Adapted from The Structure of the Human Brain, a photographic atlas. DeArmond, 1989 Oxford Publishing Limited (Academic) [86] . Reproduced with permission of the licensor through PLSclear. The license restrictions of the original publication apply to modification & reuse.

ID-25: Rubrocerebellar tract

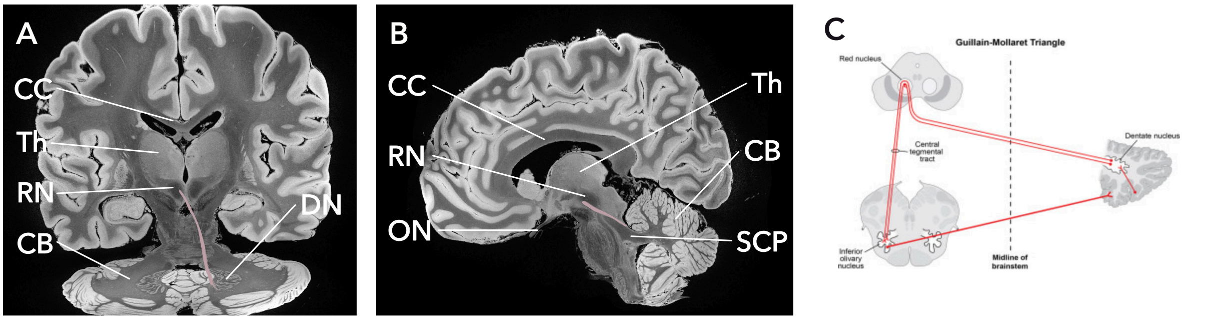

Figure S22. **A** Coronal section, posterior view of left rubro-cerebellar tract (rcet) displayed in red. **B** Parasagittal section, view from left. **C** Illustration of the so-called Guillan-Mollaret triangle (dentatorubroolivocerebellar circuit) by [88]. According to which, axons originating from the dentate nucleus reciprocally project to the contralateral red nucleus. V2-tract also describes this pathway see A. Reprinted from [88] with permission from Elsevier. The license restrictions of the original publication apply to modification & reuse.

ID-26: Rubroolivary tract

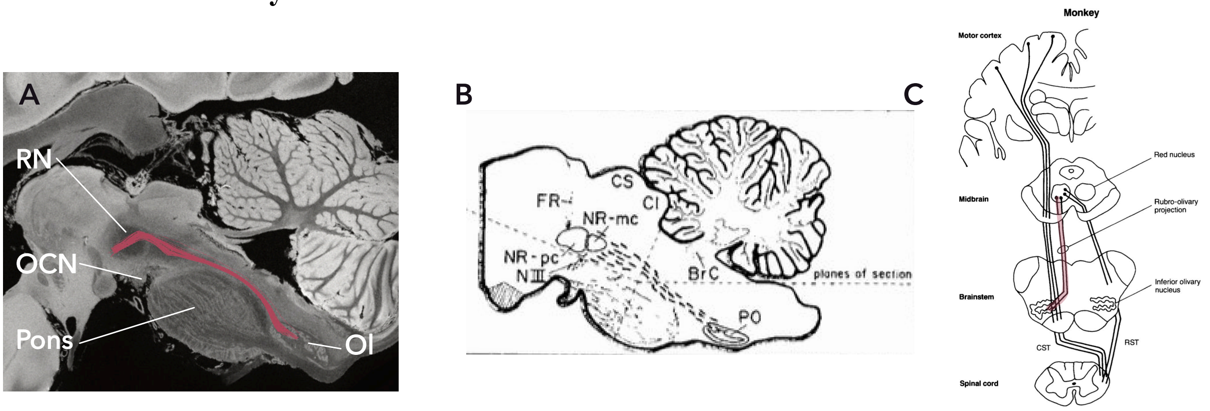

Figure S23. **A** Parasagittal section, left V2-Rubro-olivary tract (rot) displayed in pink. **B** Schematic drawing of degenerated rubro-olivary fibres as seen after lesioning the RN in monkeys [89]. Arrangement and orienation of fibres seem to be congruent to V2-tract. **C** Scheme adapted from [90] which shows section of a monkey brain and illustrates the rot (highlighted in pink). Illustrations for B are used with permission of John Wiley & Sons – Books from [89] permission conveyed through Copyright Clearance Center, Inc. The license restrictions of the original publication apply to modification & reuse. Reprinted from [90] with permission from Elsevier. The license restrictions of the original publication apply to modification & reuse.

IDs-27-30: Subthalamic nucleus – Brodman area 1-3 / 4 / 6 / 8

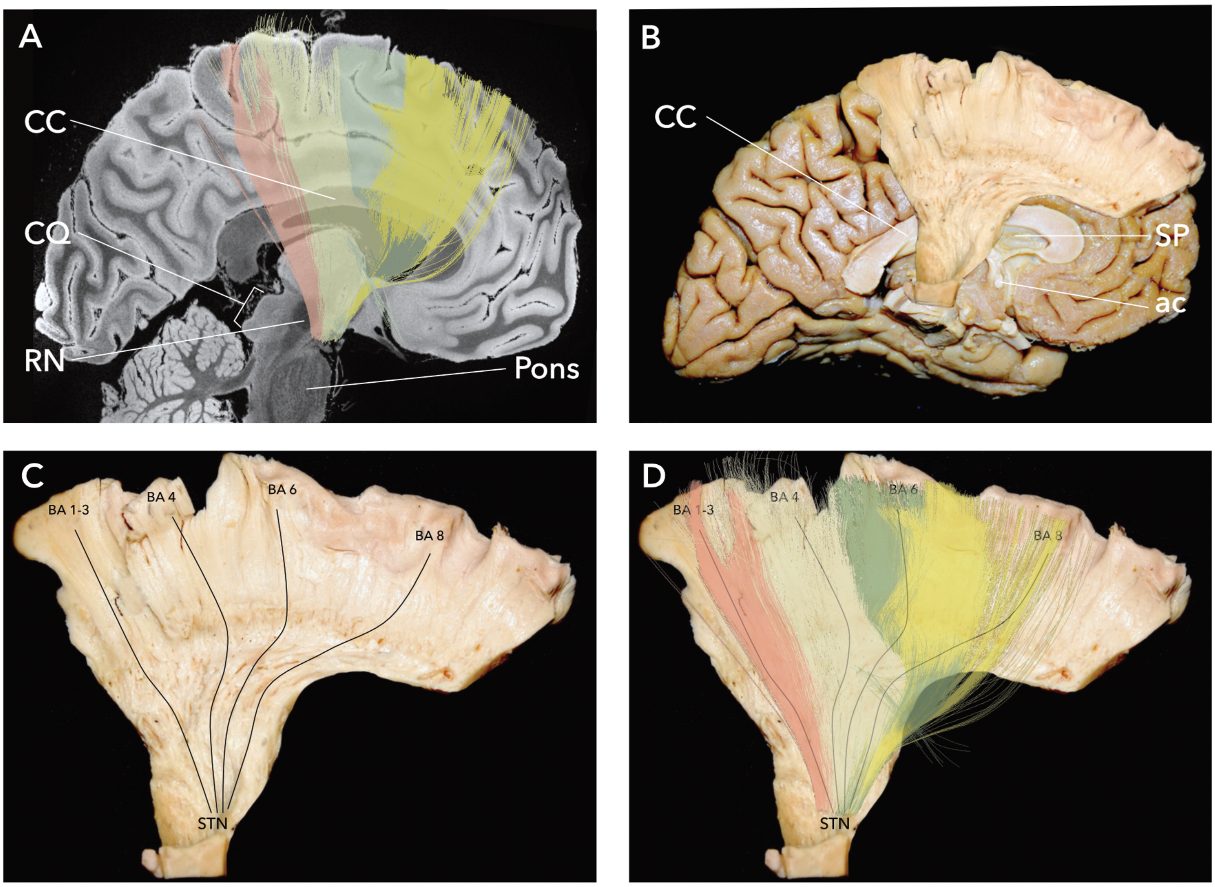

Figure S24. A Parasagittal section, view from right. Red: STN-BA1-3-fibres, light yellow: STN-BA4-fibres, green: STN-BA6-fibres, yellow: STN-BA8-fibres. **B** Corresponding fibre dissection superimposed to hemisphere for better understanding of spatial relations. **C** Fibre dissection of STN-BA1-3-fibres, STN-BA4-fibres, STN-BA6-fibres and STN-BA8-fibres. **D** Superimposed V2-tracts seem to show similar configurations. B-D Specimens kindly contributed by Georgios Skandalakis.

ID-31: Subthalamic nucleus – Brodman area 10

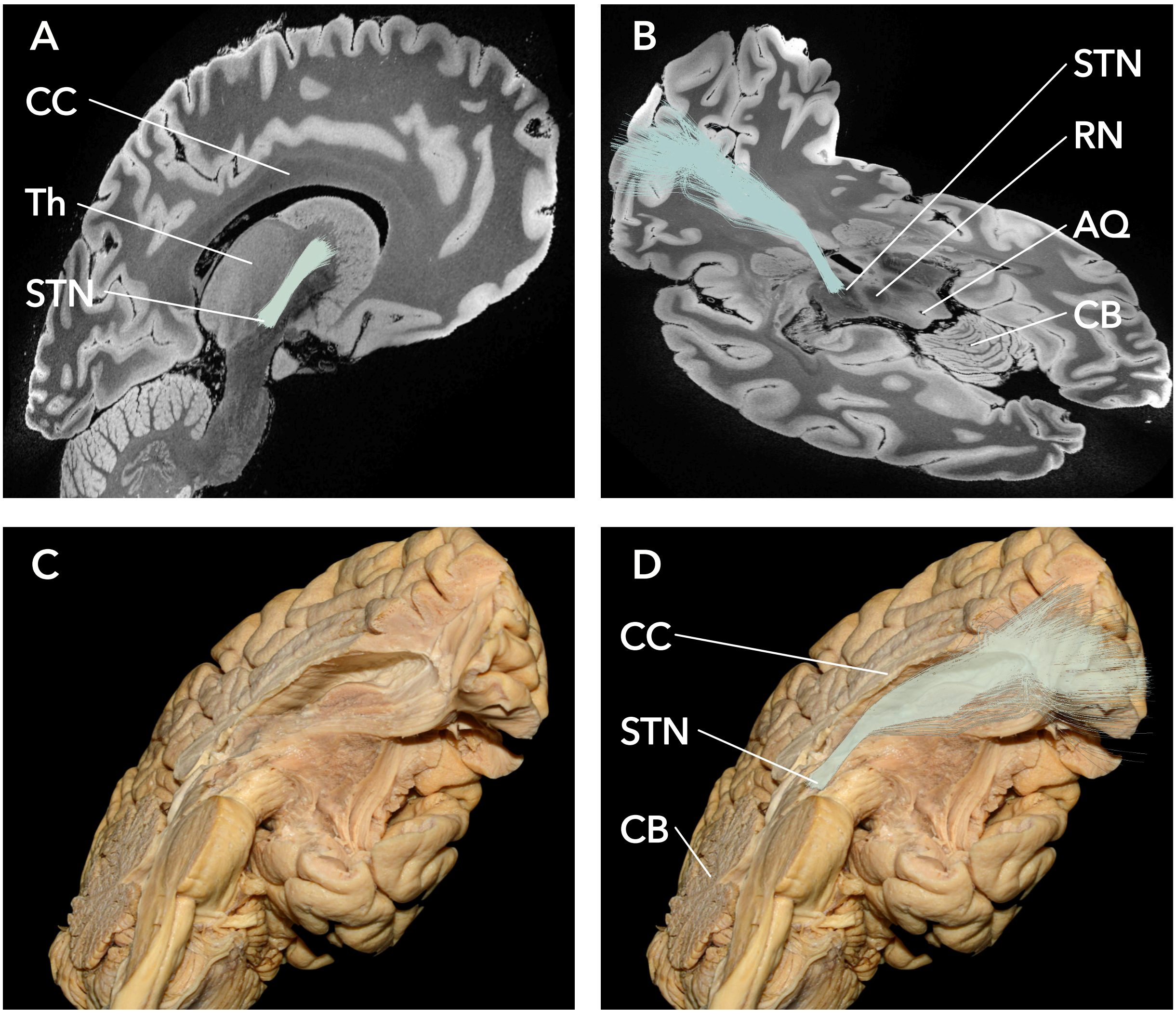

Figure S25. A Parasagittal section, view from right. Left STN-BA10-fibres shown in pastel green. **B** Combined axial and coronal section for better spatial understanding, posterosuperior view. **C** White matter dissection kindly provided by Georgios Skandalakis. **D** V2-tracts indicate comparable extents.

ID-35: Subthalamic nucleus – Brodman area 45/47

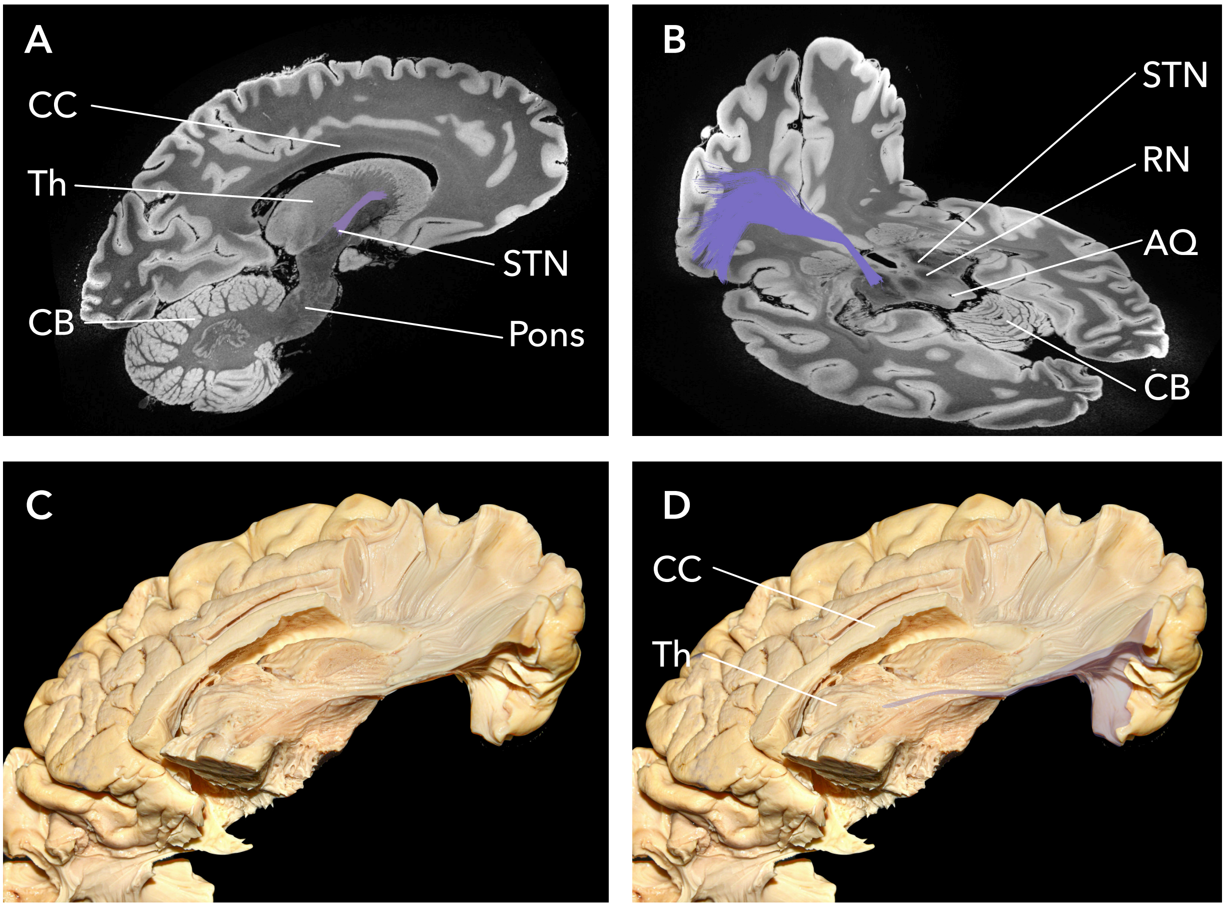

Figure S26. A Parasagittal section, view from right. Left STN-BA45/47-fibres shown in pastel purple. **B** Combined axial and coronal section for better spatial conception, posterosuperior view. **C** Inferior-medial view of white matter dissection kindly provided by Georgios Skandalakis. **D** STN-Ba 45/47 highlighted in purple.

Section S4. Validation studies of multitract model and Cleartune algorithm

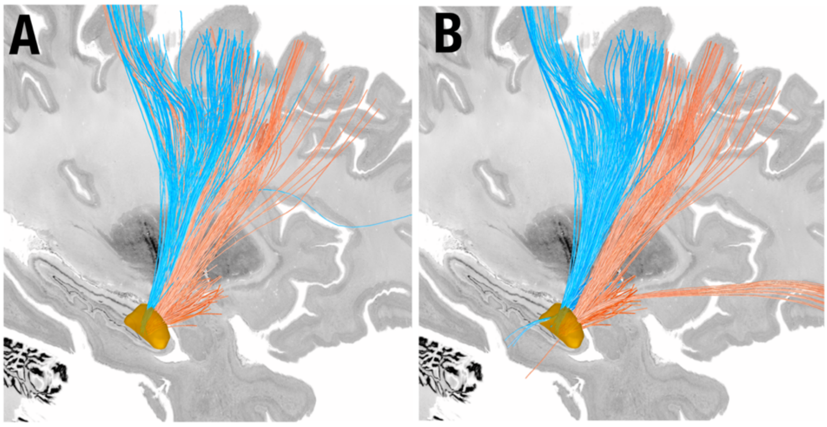

Figure S27. **Bradykinesia and rigidity results.** A) Bradykinesia and rigidity tracts as defined by the original model are visualized together. B) Regressing out bradykinesia improvements from rigidity improvements and vice versa does not show qualitative changes on an anatomical level.

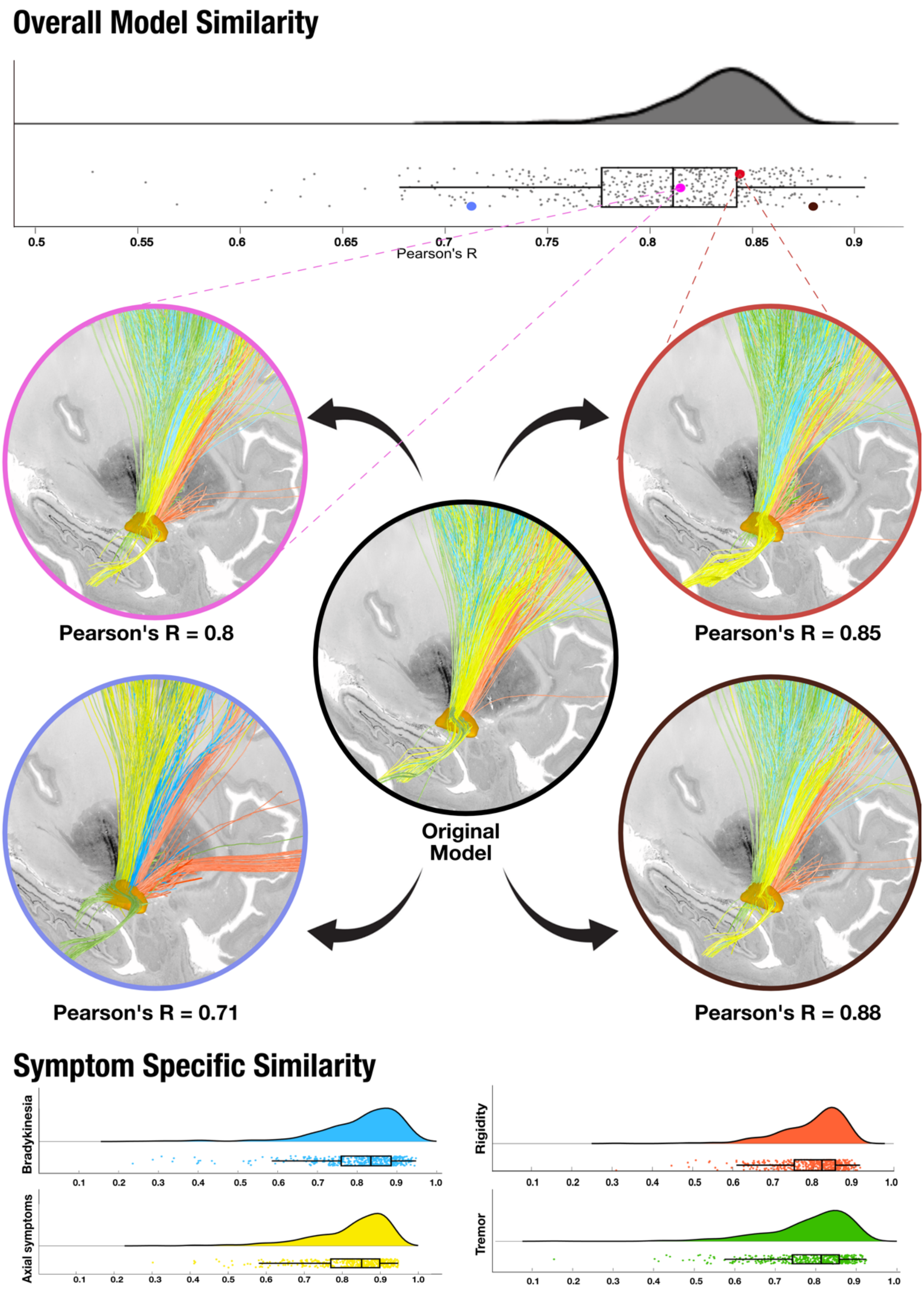

Overall Model Similarity
0.5
0.55
0.6
0.65
0.7
0.75
0.8
0.85
0.9
Pearson's R
Pearson's R = 0.8
Pearson's R = 0.85
Original Model
Pearson's R = 0.71
Pearson's R = 0.88
Symptom Specific Similarity
Bradykinesia
Rigidity
Axial symptoms
Tremor
0.1
0.2
0.3
0.4
0.5
0.6
0.7
0.8
0.9
1.0

Figure S28. To test robustness of model results as a function of spatial uncertainty in stimulation sites, we recalculated the model 1,000 times, each time after spatially jittering electric fields based on a 3D Gaussian distribution with 2 mm full width half maximum. Resulting models were highly similar (correlations of fiber weightings across fibers are shown in the figure, four example models are shown). Mean Pearson R_{UPDRS}= 0.80 ± 0.057; Mean Pearson $R_{Bradykinesia}$ = 0.80 ± 0.12; Mean Pearson $R_{Rigidity}$ = 0.79 ± 0.08; Mean Pearson $R_{AxialSymptom}$ = 0.82 ± 0.10; Mean Pearson R_{Tremor} = 0.78 ± 0.10). Source data are provided as a Source Data file.

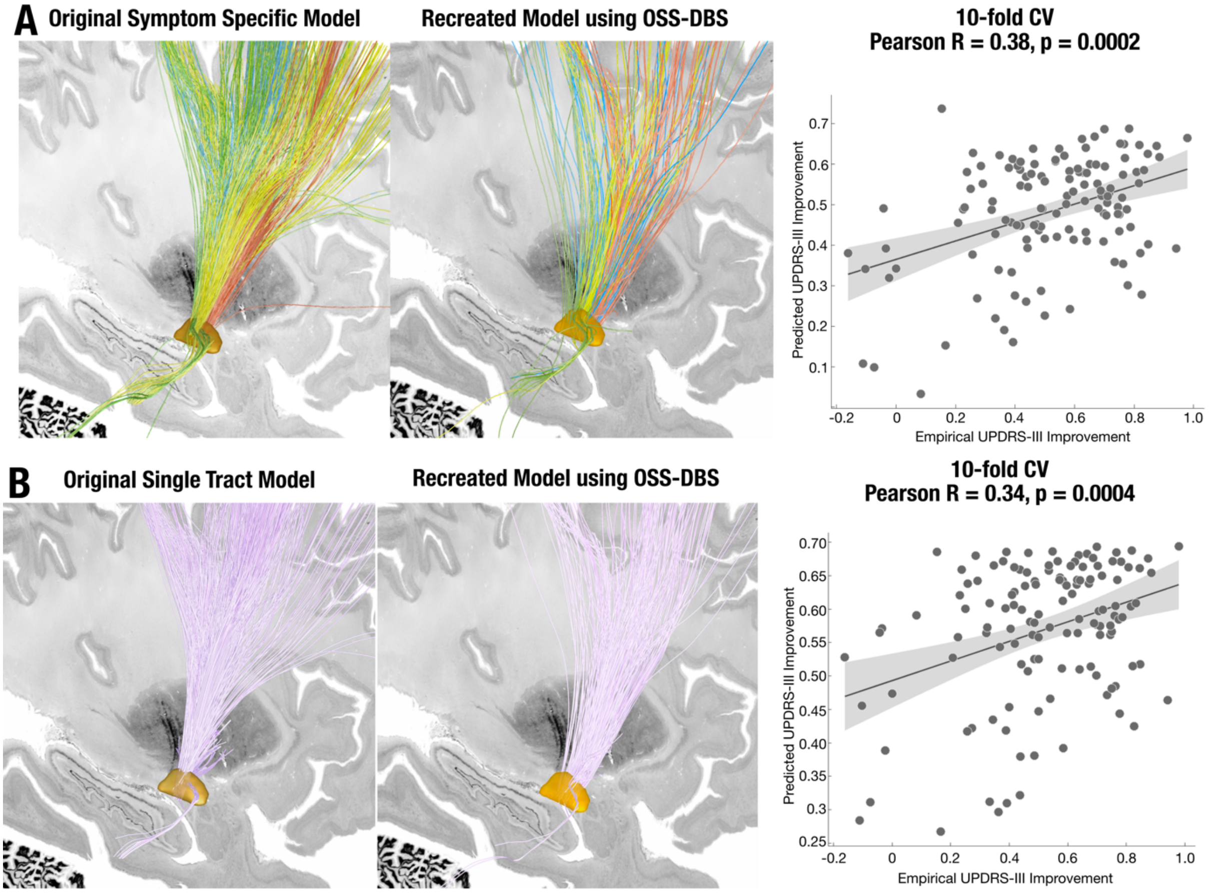

Figure S29. Comparision of symptom specific model calculate using Lead-DBS and OSS-DBS. Second, we aimed to rule out that our results would be specific to the processing pipeline used for biophysical modelling (FieldTrip / SimBio pipeline as adapted for Lead-DBS). Thus, we recomputed results using a more elaborate pipeline that was independently created by a different team of researchers, OSS-DBS [91]. The resulting model shared a similar topography, and similar in-sample cross validation results ($R_{multitract}$ = 0.38, p = 0.0002, MAE = 0.18 ± 0.13; $R_{singletract}$ = 0.34, p = 0.0004, MAE = 0.18 ± 0.14), as with the one created by our default pipeline. Source data are provided as a Source Data file. The line and shaded area of the correlation plots signify the general linear model at 95% confidence interval

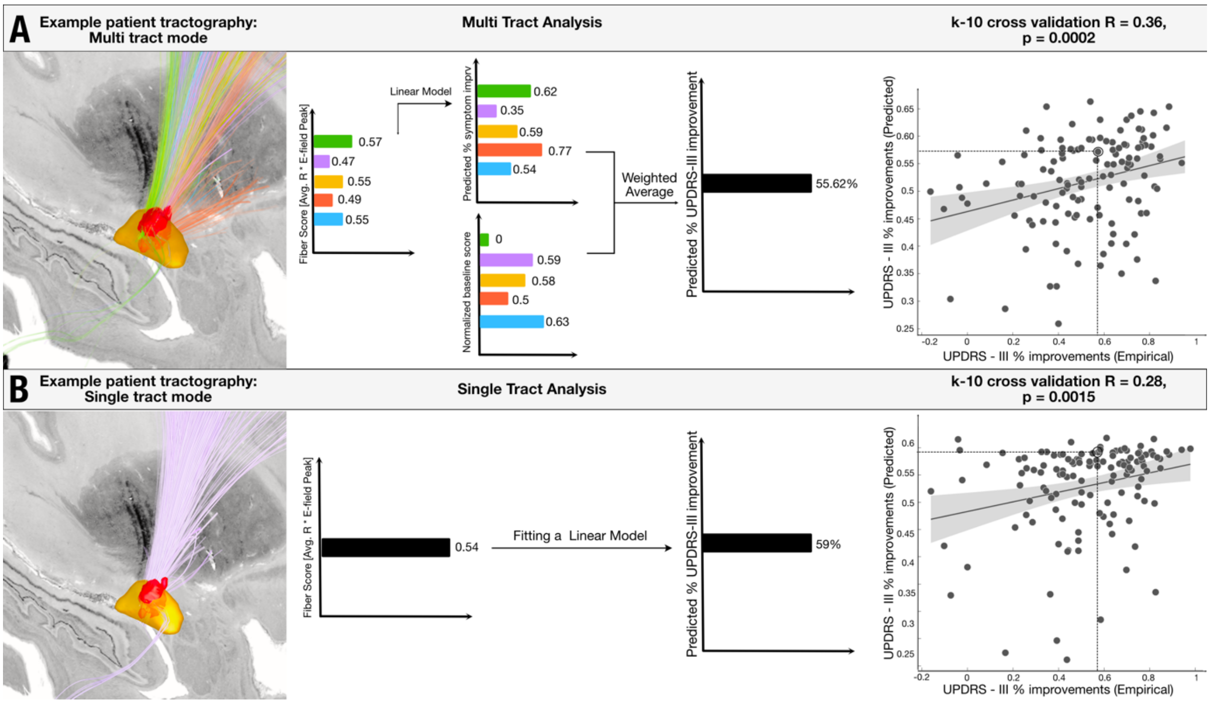

Figure S30. Multitract model considering five symptoms. Repeating the main analysis with the original cohort of (N = 129) patients, considering five symptoms (rather than four) yields significant and positive results (R = 0.36, p = 0.0002, MAE = 0.18 ± 0.13). As with the four symptom model, the five symptom model can explain larger varience in the clinical outcome when compared to the single tract analysis (R = 0.28, p = 0.0015, MAE = 0.18 ± 0.13). Source Data provided as Source Data file. The line and shaded area of the correlation plots signify the general linear model at 95% confidence interval.

During 10-fold cross-validation, sets of patients were randomly assigned to sets which were then iteratively left out. This process can be done in numerous ways (i.e., assigning different combinations of patients to folds). To rule out that the current selection was not generalizable, we repeated the process 1,000 times, each time randomly assigning patients to folds. This led to very comparable outcomes (multi-tract model, average R = 0.34, p = 0.0006; single tract model, average R = 0.20, p = 0.02). Figure S31 descibes statistics across the 1000 iterations, including error plots, and the distribution of R- and p-values across the folds.

We wanted to rule out that the choice of k (in k-fold cross validation) did not have a significant impact on our results. Therefore, we repeated the analysis with 5-fold and 7-fold cross validation which again yielded similar results for both the multi-tract (R = 0.37, p = 0.0002 and

R = 0.36, p = 0.0002, respectively) and single tract model (R = 0.35, p = 0.0004 and R = 0.27, p = 0.002).

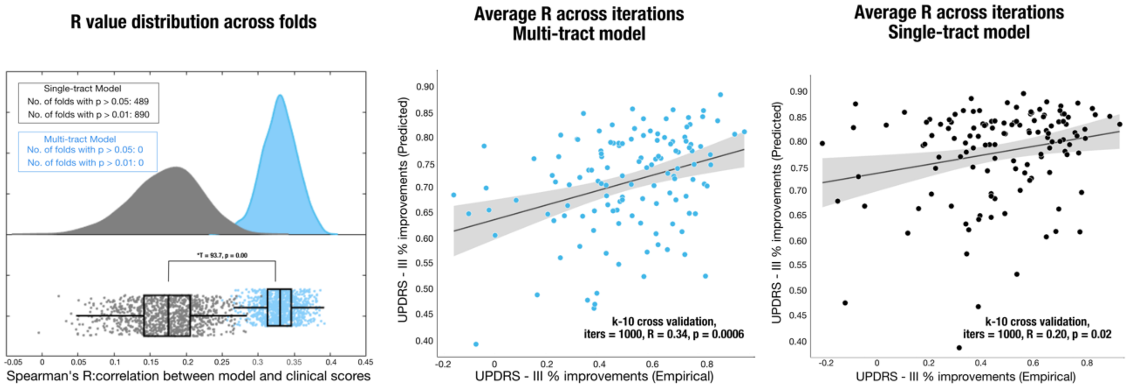

Figure S31. Left panel: The greatest density of correlation values for 10-fold cross-validations falls in the range between 0.3 to 0.4 for the multi-tract model and between 0.25 to 0.3 in the single-tract model (Average $R_{multitract}$ = 0.33 ± 0.02; Average $R_{singletract}$ = 0.18 ± 0.04). No folds have nonsignificant results calculated at p > 0.05 in the multi-tract model but 9 folds with p > 0.05 and 375 folds with p > 0.01 in the single-tract model. **Right panel:** 10-fold cross validation estimates averaged across 1000 iterations both models ($R_{multitract}$ = 0.34, p = 0.0006, MAE = 0.18 ± 0.13; $R_{singletract}$ = 0.20, p = 0.02, MAE = 0.18 ± 0.14). The line and shaded area of the correlation plots signify the general linear model at 95% confidence interval.

Correlation plots - out of sample cross validation on TWEED dataset

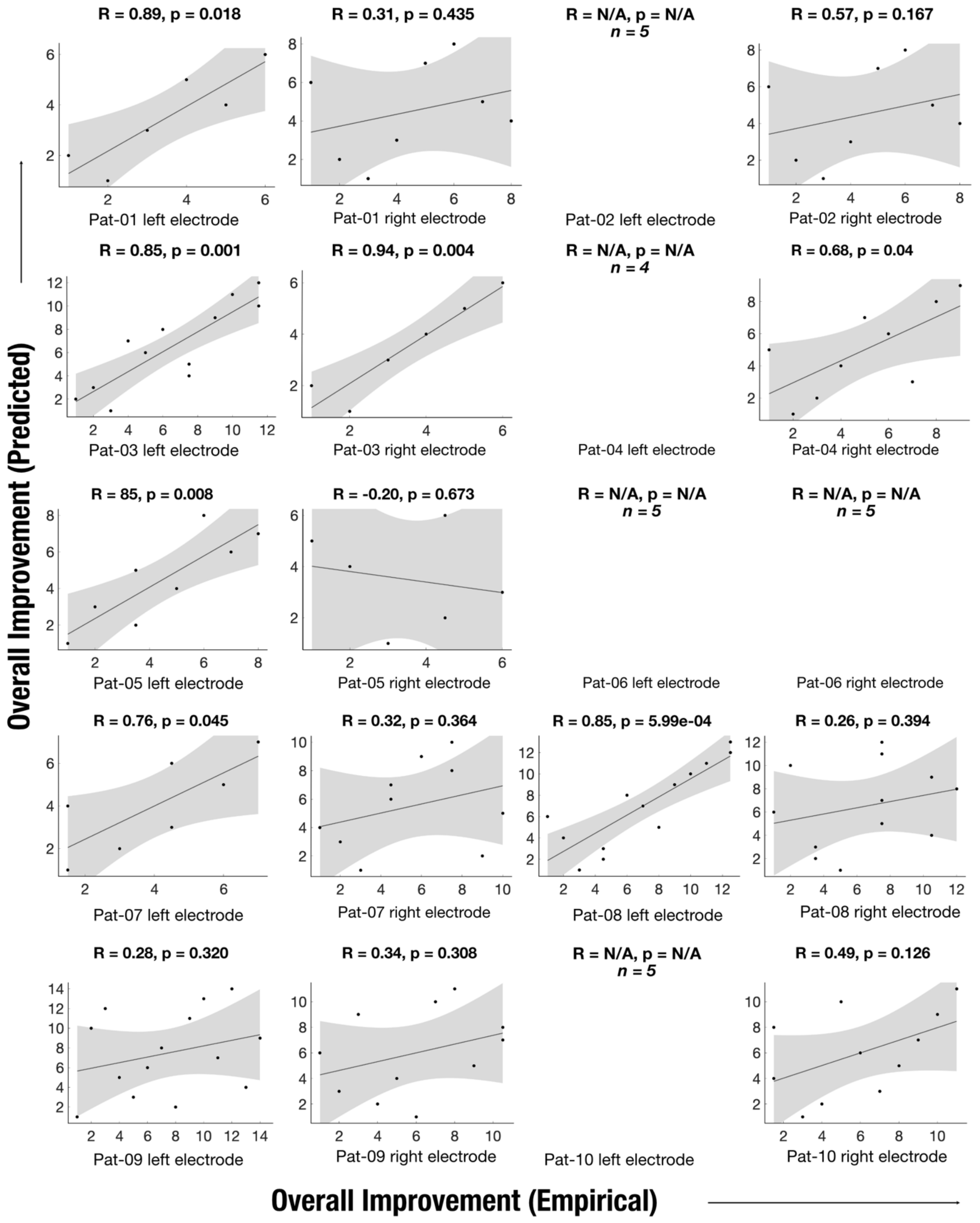

Figure S32. Correlation plots between ranks of UPDRS-III improvements estimated based on the original model and each of the 20 electrodes tested in the TWEED dataset with more than six stimulation settings are shown here. N = 6 of the electrodes were only tested with 4, 5, 5, 5, 5 settings, respectively. For these, correlation values were not calculated given the low number of data points. The line and shaded area of the correlation plots signify the general linear model at 95% confidence interval. Source data available as source data file.

Section S5. Feasibility trial for prospective application of Cleartune in a clinical setting.

To test the feasibility of applying Cleartune in a clinical setting, a feasibility trial was carried out in a small sample of n=5 prospective patients. This trial was designed to include a randomization step, where the patient was blinded to the administration of Cleartune vs. clinical settings. Clinical data, which included the pre-operative T1w, T2w, and post operative CT images was used to localize DBS electrodes in each patient. Baseline scores were taken in the stimulation and medication off states. The Cleartune algorithm was executed for each electrode separately, for 500 iterations each. This led to Cleartune suggested stimulation settings, which were stored in the pulse generator as an additional program to the existing standard of care (SoC) setting. In the second week, Cleartune settings or clinical settings were applied in randomized order, each for 24 hours. Resulting UPDRS-III scores were taken after 24 hours and the respective other program was switched on to be evaluated after another 24 hours. Figure S33 summarizes the trial design. Results are documented in table S5. In multiple cases, Cleartune suggested higher amplitudes than tolerable, and were hence reduced by the clinical team (without altering contact choices). Table S5 reports both suggested and programmed amplitudes. From a baseline of 49.8 ± 22.1 UPDRS-III points, under Cleartune settings, scores improved by 34.4 ± 13.1 points (73 ± 11.8%). Under standard of care settings, scores improved by 31.8 ± 15.1 points (65.4 ± 12.1%). In four of the five patients, Cleartune settings led to a higher improvement than SoC settings. In the fourth patient, improvements were comparable (36 vs. 38 points improvement). While three of the five patients preferred Cleartune over SoC settings, in two patients, Cleartune settings led to side-effects (dyskinesia in patient 05 and dizziness in patient 04). While generally promising, given the low N, these results should not be overinterpreted. Rather, this trial was carried out to test feasibility of applying Cleartune in a clinical setting and to gather first experience in

preparation for a proper prospective trial. As such, the trial was not powered to compare Cleartune vs. SoC settings (non-inferiority or superiority).

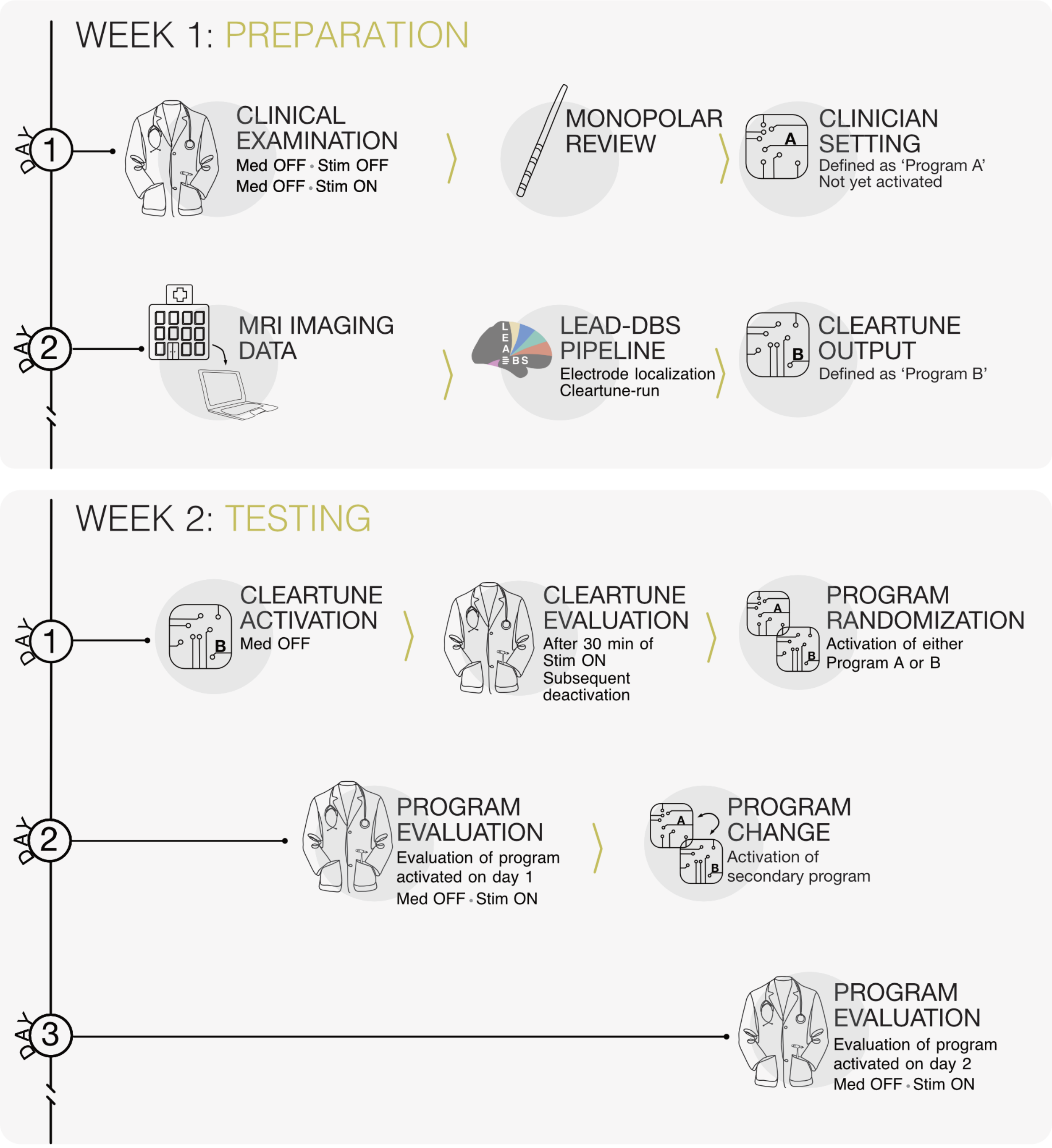

Figure S33. Timeline of prospective feasibility study. In week 1, clinical examinations (MDS-UPDRS-III scores) were carried out in Med OFF / Stim OFF as well as Med OFF / Stim ON states. A monopolar review was carried out to derive at the clinicial setting (standard of care, settings were stored as Program A in the pulse generator).

Later in the same week, asynchronously (here shown on "Day 2" for simplicity), the MRI and CT imaging data were used to localize electrodes using Lead-DBS and fed into the cleartune algorithm together with the baseline Med OFF / Stim OFF scores (to weight predominant tremor, bradykinesia, rigidity or axial symptoms in patient personalized fashion). In week 2, on the first day, the stimulation parameters suggested by Cleartune were briefly switched on for 30 minutes only to rule out side effects. The amplitude suggested by Cleartune was lowered below the threshold in case of side effects and stored as Program B in the pulse generator. Finally, in randomized fashion, either Program A or B were activated. On Day 2, the respective active program was evaluated (MDS-UPDRS-III in Med OFF / Stim ON state). The respective other program was then activated and evaluated in the same fashion on Day 3.

Table S4. Feasibility study of applying Cleartune in a clinical context

	**Cleartune [%]**			**Standard of Care [%]**		
**Patient**	**Settings RH**	**Settings LH**	**Global**	**Settings RH**	**Settings LH**	**Global**
Patient - 01	3: 44.1%; 7: 30.6%; 8: 25.3%, Amp: 3mA [5 mA]	3: 100%, Amp: 2.1 mA [3 mA]	81.25 [B: 86.67%, R: 100%, A: 50%, T: N/A]	3: 50%; 4: 50%, Amp: 2.4 mA	3: 33.33%; 4: 33.33%; 5: 33.33%, Amp: 1.5 mA	62.50 [B: 53.33%, R: 87.5%, A: 37.5%, T: N/A]
Patient - 02	1: 7.5%; 4: 7.5%; 5: 10%; 7: 37.5%; 8: 37.5%, Amp: 3.5 mA [5 mA]	1: 100%, Amp: 3.3 mA	63.63 [B: 69.5%, R: 30%, A: 63.6%, T: N/A]	3: 50%; 4: 50%, Amp: 3.1 mA	3: 50%; 4: 50%; Amp: 3.2 mA	56.81 [B: 43.5%, R: 70%, A: 45.4%, T: N/A]
Patient - 03	1: 31.2%; 4: 6.63%; 7: 44.3%; 8: 17.9%, Amp: 3.5 mA [5 mA]	1: 60%; 4: 8.1%; 8: 32.0%, Amp: 3.0 mA [5 mA]	60.60 [B: 78.94%, R: 42.8%, A: 44.4%, T: N/A]	2: 60%; 5: 40%, Amp: 3.0 mA	2 to 7:15%, Amp: 2.5 mA	54.54 [B: 78.94%, R: 14.3%, A: 44.4%, T: N/A]
Patient - 04	4: 58%; 8: 42%, Amp: 5 mA	1: 22%; 8: 78%; Amp: 3.06 mA	55.29 [B: 50%, R: 6.67%, A: 71.4%, T: 100%]	2 - 4: 50%, 5 - 7: 50%, Amp: 3.0 mA	1: 20%; 2,3,4: 80%, Amp: 3.0 mA	57.64 [B: 59.4%, R: 20%, A: 60.7%, T: 100%]
Patient - 05	2: 41.7%, 3: 4.7%, 4: 6.05%, 5: 47.5%, Amp: 3.1 mA	1: 38.1%, 4: 57%, 5: 3.58%, Amp: 3.4 mA	90 [B: 100%, R: 100%, A: 50%, T: 100%]	2: 100%, Amp: 1.9 mA	3: 50%, 4:50%, Amp: 2.1 mA	85 [B: 85.7%, R: 100%, A: 60%, T: 100%]

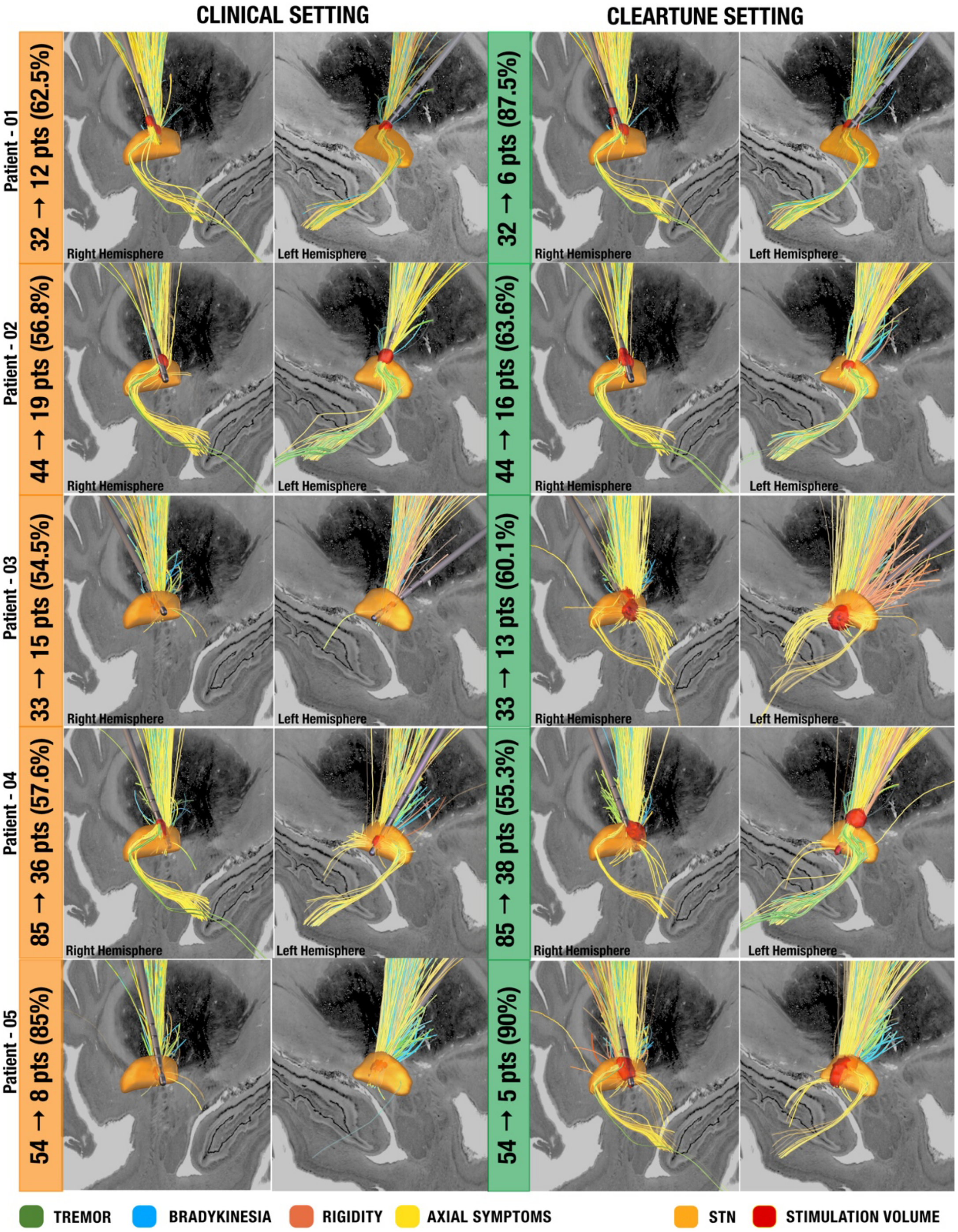

Figure S34. Feasibility Study of Cleartune The stimulation volume programmed by standard of care settings compared with the stimulation volume programmed by cleartune. Fibers tractography in each patient is weighted by the strength of connection to the stimulation volume.

Table S5. Statistics of clinical scores and sub scores across cohorts.

Symptom	UPDRS-III/MDS items	Mean and standard deviation of baseline clinical scores
**Bradykinesia**	23 (Finger tap R and L)<br>24 (Toe tap R and L)<br>25 (Hand move R and L)<br>26 (Leg agility R and L)<br>and MDS – Toe tapping (R and L).	**Baseline:**<br>17.2 ± 7.00<br>**Improvement:**<br>44.99 ±32.98%
**Rigidity**	22 (rigidity neck, rigidity arm R and L, rigidity leg R and L).	**Baseline:**<br>9.30 ± 5.10<br>**Improvement:**<br>70.40 ± 28.33%
**Tremor**	20 (tremor rest arm R and L, tremor rest leg R and L)<br>21 (action tremor hand R and L)/MDS (kinetic tremor hand R and L)<br>MDS tremor (lip/jaw)<br>MDS constancy of rest tremor.	**Baseline:**<br>4.58 ± 5.24<br>**Improvement (thresholded):**<br>57.24 ± 42.38%
**Axial**	27 (Arise chair)<br>28 (posture)<br>29 (gait)<br>30 (postural stability)<br>31 (Body bradykinesia)<br>MDS-Freezing of Gait (FoG)	**Baseline:**<br>10.0 ± 4.71<br>**Improvement:**<br>46.93 ± 30.24%

Section S6. Modelling Considerations

A natural question that may arise is the validity of a model given the relatively low correlation coefficients (R), and consequentially, R2 values. To discuss this question, we would like to raise the following points (Fig S35).

1. First, we need to ask how much variance DBS modeling should at best explain. Clinical improvements are not just based on electrode placement and stimulation volumes, but governed by many factors, such as disease subtype, age, sex, levodopa response, duration of disease, comorbid other conditions, etc. We estimate that these factors alone will explain ~50% of DBS response[92].
2. Noise in clinical scores (inter- and intra-rater test-retest reliability of the UPDRS-III) will account for another 15%[93].
3. Imaging resolution and electrode placement & modeling inaccuracy may explain another 15-20% of variance[94–96].
4. The use of multi-site datasets such as in the present study may add residuals that may explain another 5-10% of the variance.

Based on this assessment, we believe that ~10% explained variance (R values of ~0.3) may realistically be expected in a multi-site dataset such as the present one. However, we believe that such models are still useful because electrode placement / stimulation settings are the only factor that can be influenced, whereas other components such as patient age, disease type and disease onset are immutable. Therefore, identification of a robust, and highly optimal target that can only explain ~10% of variance would still remain an important finding in our field.

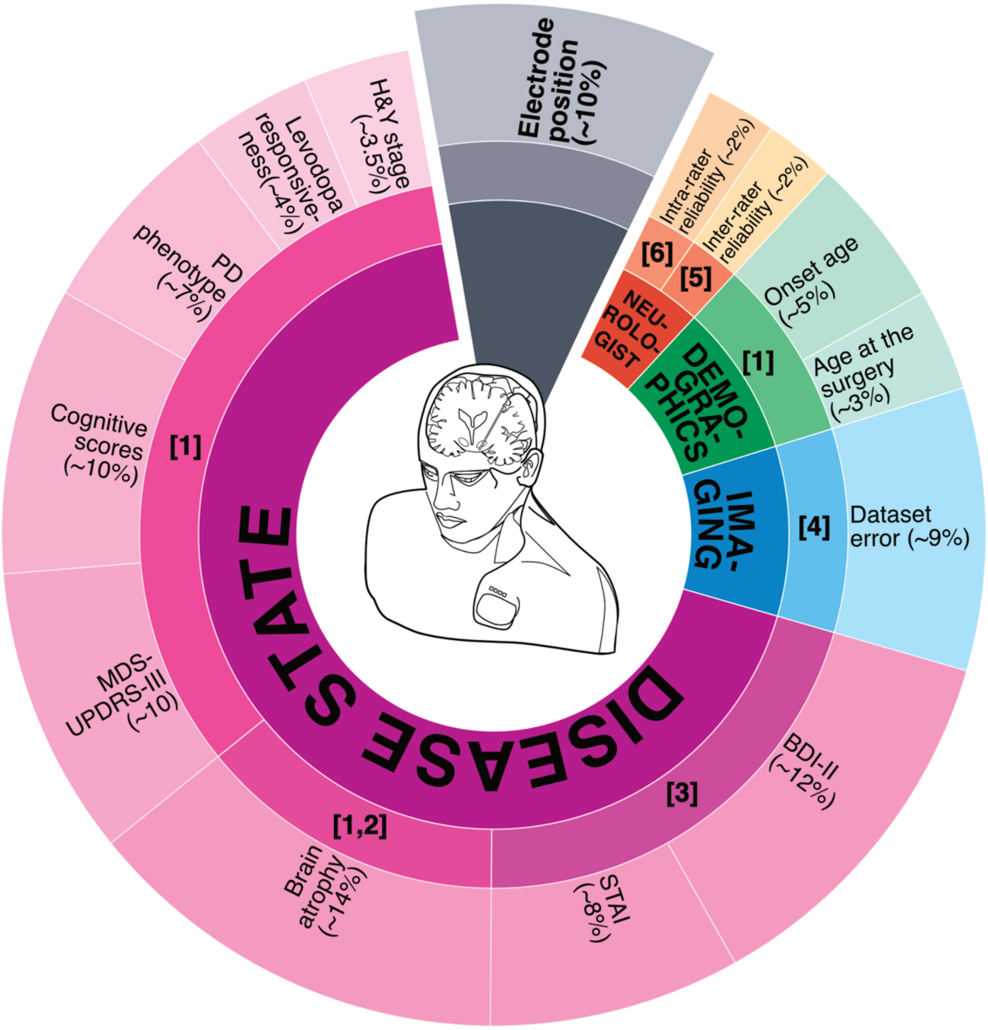

Figure S35. Modeling considerations. The pie chart is illustrative of the various factors that influence the outcome of the patient undergoing DBS. With this, we could conclude that the maximum impact electrode positioning can have on the patient outcome is ~10%.

Section S7. Cleartune Algorithm description

Cleartune is a computational tool to optimize stimulation protocols trained on the multi tract model. The optimization problem for our study is formulated as

$$\mathbf{J} = \operatorname{argmax} \sum_{s=1}^{S} \omega_s \hat{\imath}_s(J) \text{ such that } \begin{cases} \|\mathbf{J}\|_1 \leq 5 \text{ mA} \\ -4 \text{ mA} \leq Jc \leq 0 \text{ for } c = 1 \text{ to } c = N \text{ contacts} \end{cases}$$

where S defines specific symptoms, ω_s is the symptom weight and $\hat{\imath}_s$ is the estimated symptom improvement for stimulation J is composed of currents across each contact J_c. For conventional DBS electrodes (4-8 contacts), the parameters space to be investigated by a FEM based volume conductor is relatively large (≈ 1 min per sample). Therefore, we employ a surrogate optimizer (see surrogateopt in MATLAB) based on interpolation of radial basis functions through sparsely and randomly sampled parameter space. In brief, the algorithm performs the following steps

**Cleartune Algorithm**
1. Set optimizer parameters, e.g. the maximum number of samples, the minimum number of random samples to create a surrogate model, the objective limit, etc.<br>2. Define current bounds (see Eq. 1)<br>3. Initiate $\mathbf{J_{init}}$<br>4. Solve the FEM problem for $\mathbf{J_{init}}$. If necessary, solve the additional random samples within the current bounds to create a surrogate model.<br>5. Investigate the parameter space using the surrogate model<br>a. Randomly sample around the incumbent (the best yet observed) using a merit function that balances exploration of the parameter space and minimization of the surrogate. The sample with the smallest value is the adaptive sample.<br>b. Solve the FEM problem for the adaptive sample and refine the surrogate.<br>c. Update incumbent if a new global minimum (i.e., minimum error or maximum value) is observed.<br>6. Update the sampling dispersion depending on the rate of success of adaptive samples against incumbents.<br>7. If convergence is reached before the maximum number of iterations are run, but the evaluated value is still lower than the objective value, reset by discarding all adaptive points.

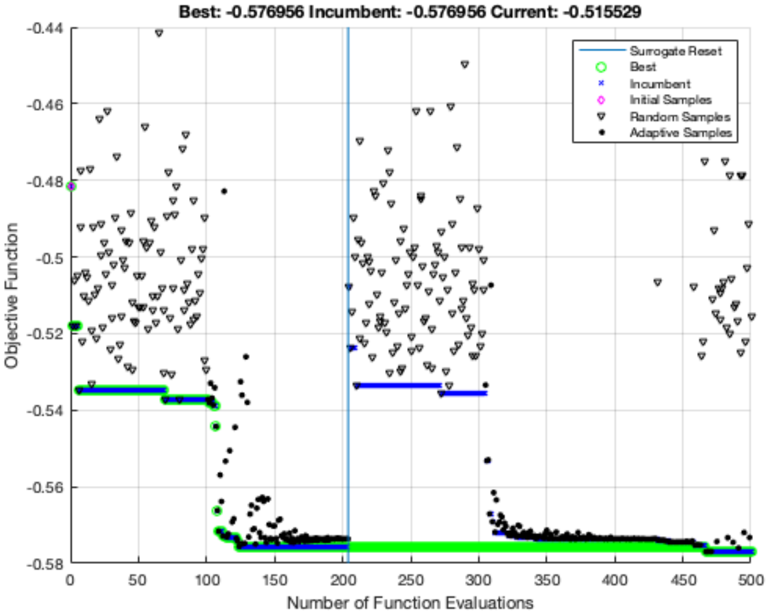

Figure S36: Example of an optimization run in Cleartune. Only samples computed with the FEM model are shown. The algorithm is initialized to calculate the stimulation volume around the 3rd contact of the electrode. An initial model is calculated with the first 100 random samples. Once the model is constructed, adaptive sampling is performed (black dots) where the model searches for a local minimum phase. At the 200th iteration, the surrogate model is reset which implies that a new surrogate model is constructed. The purple points represent the best values since the previous surrogate reset. This cycle of adaptive sampling and random sampling is continued until the number of iterations equal the maximum functional evaluation set externally by the user or if the objective function reaches the desired value. In this case, though the objective function value is not reached, 500 iterations have been completed and therefore, the optimization is completed.

For the current application, we initialized the optimization protocol to calculate the stimulation volume at the 3rd electrode contact. However, this is a user defined parameter and therefore, the user has the ability to simulate a monopolar review. Next, 120 random samples are taken to define the surrogate model for the further adaptive sampling (Fig. S36). The maximum number of FEM samples is limited to 500, based on observation that most FEM calculations converge at around ~450 iteration mark. The objective limit is set to 0.9, which corresponds to 90% improvement. If the local convergence did not fulfil the objective limit criterion, the model is reset.

Supplementary references

1. Husch, A., Petersen, M. V., Gemmar, P., Goncalves, J. & Hertel, F. PaCER - A fully automated method for electrode trajectory and contact reconstruction in deep brain stimulation. *Neuroimage Clin* **17**, 80–89 (2017).
2. Lofredi, R. *et al.* Interrater reliability of deep brain stimulation electrode localizations. *NeuroImage* **262**, 119552 (2022).
3. Geng, X. *et al.* Intra-operative characterisation of subthalamic oscillations in Parkinson's disease. *Clinical Neurophysiology* **0**, (2018).
4. Horn, A., Neumann, W.-J., Degen, K., Schneider, G.-H. & Kühn, A. A. Toward an electrophysiological "sweet spot" for deep brain stimulation in the subthalamic nucleus. *Hum Brain Mapp* (2017) doi:10.1002/hbm.23594.
5. van Wijk, B. C. M. *et al.* Localization of beta and high-frequency oscillations within the subthalamic nucleus region. *Neuroimage Clin* **16**, 175–183 (2017).
6. Neumann, W.-J. *et al.* A localized pallidal physiomarker in cervical dystonia. *Ann Neurol* (2017) doi:10.1002/ana.25095.
7. Lofredi, R. *et al.* Dopamine-dependent scaling of subthalamic gamma bursts with movement velocity in patients with Parkinson's disease. *Elife* **7**, e31895 (2018).
8. Rappel, P. *et al.* Theta-alpha oscillations characterize emotional subregion in the human ventral subthalamic nucleus. *Mov. Disord.* **10**, 672–7 (2019).
9. Al Awadhi, A. *et al.* Electrophysiological confrontation of Lead-DBS-based electrode localizations in patients with Parkinson's disease undergoing deep brain stimulation. *NeuroImage: Clinical* **34**, 102971 (2022).
10. Boëx, C. *et al.* Validation of Lead-DBS β-Oscillation Localization with Directional Electrodes. *Bioengineering* **10**, 898 (2023).

11. Horn, A. & Kühn, A. A. Lead-DBS: a toolbox for deep brain stimulation electrode localizations and visualizations. *NeuroImage* **107**, 127–135 (2015).
12. Xu, Y. *et al.* Deep Brain Stimulation Electrode Reconstruction: Comparison between Lead-DBS and Surgical Planning System. *JCM* **12**, 1781 (2023).
13. Sitz, A. *et al.* Determining the orientation angle of directional leads for deep brain stimulation using computed tomography and digital x-ray imaging: A phantom study. *Med. Phys.* **44**, 4463–4473 (2017).
14. Dembek, T. A. *et al.* Directional DBS leads show large deviations from their intended implantation orientation. *Parkinsonism Relat. Disord.* (2019) doi:10.1016/j.parkreldis.2019.08.017.
15. Sauner, D. *et al.* Multimodal Localization of Electrodes in Deep Brain Stimulation: Comparison of Stereotactic CT and MRI with Teleradiography. *Stereotact Funct Neurosurg* **88**, 253–258 (2010).
16. Dembek, T. A. *et al.* Temporal Stability of Lead Orientation in Directional Deep Brain Stimulation. *Stereotact Funct Neurosurg* **99**, 167–170 (2021).
17. Kehnemouyi, Y. M. *et al.* Modulation of beta bursts in subthalamic sensorimotor circuits predicts improvement in bradykinesia. *Brain* **12**, E33-14 (2020).
18. Segura-Amil, A. *et al.* Programming of subthalamic nucleus deep brain stimulation with hyperdirect pathway and corticospinal tract-guided parameter suggestions. *Human Brain Mapping* hbm.26390 (2023) doi:10.1002/hbm.26390.
19. Butenko, K. & van Rienen, U. DBS imaging methods III: Estimating the electric field and volume of tissue activated. in *Connectomic Deep Brain Stimulation* 147–168 (Elsevier, 2022).

20. Ewert, S. *et al.* Optimization and comparative evaluation of nonlinear deformation algorithms for atlas-based segmentation of DBS target nuclei. *NeuroImage* 1–13 (2018) doi:10.1016/j.neuroimage.2018.09.061.

21. Vogel, D. *et al.* Anatomical Brain Structures Normalization for Deep Brain Stimulation in Movement Disorders. *Neuroimage Clin* 102271–36 (2020) doi:10.1016/j.nicl.2020.102271.

22. Oxenford, S. *et al. Lead-OR: A Multimodal Platform for Deep Brain Stimulation Surgery*. 2021.08.09.21261792 https://www.medrxiv.org/content/10.1101/2021.08.09.21261792v1 (2021) doi:10.1101/2021.08.09.21261792.

23. Dembek, T. A. *et al.* Sweetspot Mapping in Deep Brain Stimulation: Strengths and Limitations of Current Approaches. *Neuromodulation: Technology at the Neural Interface* **25**, 877–887 (2022).

24. Neudorfer, C. *et al.* Lead-DBS v3.0: Mapping deep brain stimulation effects to local anatomy and global networks. *NeuroImage* **268**, 119862 (2023).

25. Neumann, W.-J. *et al.* Functional segregation of basal ganglia pathways in Parkinson's disease. *Brain* **12**, 366 (2018).

26. de Almeida Marcelino, A. L., Horn, A., Krause, P., Kühn, A. A. & Neumann, W.-J. Subthalamic neuromodulation improves short-term motor learning in Parkinson's disease. *Brain* **28**, 10687–9 (2019).

27. Horn, A. *et al.* Lead-DBS v2: Towards a comprehensive pipeline for deep brain stimulation imaging. *NeuroImage* **184**, 293–316 (2019).

28. Horn, A. *et al.* Connectivity Predicts deep brain stimulation outcome in Parkinson disease. *Ann Neurol* **82**, 67–78 (2017).

29. Al-Fatly, B. *et al.* Connectivity profile of thalamic deep brain stimulation to effectively treat essential tremor. *Brain* **18**, 130 (2019).
30. Irmen, F. *et al.* Left prefrontal impact links subthalamic stimulation with depressive symptoms. *Ann Neurol* (2020) doi:10.1002/ana.25734.
31. Baldermann, J. C. *et al.* Connectivity Profile Predictive of Effective Deep Brain Stimulation in Obsessive-Compulsive Disorder. *Biological Psychiatry* **85**, 735–743 (2019).
32. Li, N. *et al.* A unified connectomic target for deep brain stimulation in obsessive-compulsive disorder. *Nat Commun* **11**, 3364 (2020).
33. Smith, A. H. *et al.* Replicable effects of deep brain stimulation for obsessive-compulsive disorder. *Brain Stimul* **14**, 1–3 (2021).
34. van der Vlis, T. A. M. B. *et al.* Ventral Capsule/Ventral Striatum Stimulation in Obsessive-Compulsive Disorder: Toward a Unified Connectomic Target for Deep Brain Stimulation? *Neuromodulation* **24**, 316–323 (2021).
35. Li, N. *et al.* A unified connectomic target for deep brain stimulation in obsessive-compulsive disorder. *Nature Communications* **11**, 3364 (2020).
36. Mosley, P. E. *et al.* A randomised, double-blind, sham-controlled trial of deep brain stimulation of the bed nucleus of the stria terminalis for treatment-resistant obsessive-compulsive disorder. *Transl Psychiatry* **11**, 1–17 (2021).
37. Johnson, K. A. *et al.* Basal Ganglia Pathways Associated With Therapeutic Pallidal Deep Brain Stimulation for Tourette Syndrome. *Biological Psychiatry: Cognitive Neuroscience and Neuroimaging* **6**, 961–972 (2021).

38. Gadot, R. *et al.* Tractography-Based Modeling Explains Treatment Outcomes in Patients Undergoing Deep Brain Stimulation for Obsessive Compulsive Disorder. *Biological Psychiatry* S0006322323000458 (2023) doi:10.1016/j.biopsych.2023.01.017.

39. Shang, R., He, L., Ma, X., Ma, Y. & Li, X. Connectome-Based Model Predicts Deep Brain Stimulation Outcome in Parkinson's Disease. *Front Comput Neurosci* **14**, 103–9 (2020).

40. Zhang, D. *et al.* Electrode Reconstruction Assists Postoperative Contact Selection in Deep Brain Stimulation. *World Neurosurg* **125**, 1–6 (2019).

41. Zhang, F. *et al.* Relationship between electrode position of deep brain stimulation and motor symptoms of Parkinson's disease. *BMC Neurol* **21**, 122 (2021).

42. Petry-Schmelzer, J. N. *et al.* Non-motor outcomes depend on location of neurostimulation in Parkinson's disease. *Brain* **35**, 5083–13 (2019).

43. Roediger, J. *et al.* Automated deep brain stimulation programming based on electrode location: a randomised, crossover trial using a data-driven algorithm. *The Lancet Digital Health* S258975002200214X (2022) doi:10.1016/S2589-7500(22)00214-X.

44. Petersen, M. V. *et al.* Holographic Reconstruction of Axonal Pathways in the Human Brain. *Neuron* **104**, 1056-1064.e3 (2019).

45. Morel, A. *Stereotactic Atlas of the Human Thalamus and Basal Ganglia*. (CRC Press, 2007).

46. Gallay, M. N., Jeanmonod, D., Liu, J. & Morel, A. Human pallidothalamic and cerebellothalamic tracts: anatomical basis for functional stereotactic neurosurgery. *Brain Struct Funct* **212**, 443–463 (2008).

47. Pauli, W. M., Nili, A. N. & Tyszka, J. M. A high-resolution probabilistic in vivo atlas of human subcortical brain nuclei. *Sci Data* **5**, 180063 (2018).

48. Middlebrooks, E. H. *et al.* Neuroimaging Advances in Deep Brain Stimulation: Review of Indications, Anatomy, and Brain Connectomics. *AJNR Am J Neuroradiol* **41**, 1558–1568 (2020).
49. Hacker, M. L. *et al.* Connectivity Profile for Subthalamic Nucleus Deep Brain Stimulation in Early Stage Parkinson Disease. *Annals of Neurology* **94**, 271–284 (2023).
50. Alho, A. T. D. L. *et al.* Magnetic resonance diffusion tensor imaging for the pedunculopontine nucleus: proof of concept and histological correlation. *Brain Struct Funct* **222**, 2547–2558 (2017).
51. Mayka, M. A., Corcos, D. M., Leurgans, S. E. & Vaillancourt, D. E. Three-dimensional locations and boundaries of motor and premotor cortices as defined by functional brain imaging: A meta-analysis. *NeuroImage* **31**, 1453–1474 (2006).
52. Pijnenburg, R. *et al.* Myelo- and cytoarchitectonic microstructural and functional human cortical atlases reconstructed in common MRI space. *NeuroImage* **239**, 118274 (2021).
53. Horn, A. *et al.* Connectivity Predicts Deep Brain Stimulation Outcome in Parkinson Disease. *Ann Neurol* **82**, 67–78 (2017).
54. Al-Fatly, B. *et al.* Connectivity profile of thalamic deep brain stimulation to effectively treat essential tremor. *Brain* **142**, 3086–3098 (2019).
55. Middlebrooks, E. H. *et al.* Neuroimaging Advances in Deep Brain Stimulation: Review of Indications, Anatomy, and Brain Connectomics. *American Journal of Neuroradiology* **41**, 1558–1568 (2020).
56. Petersen, M. V. *et al.* Holographic Reconstruction of Axonal Pathways in the Human Brain. *Neuron* **104**, 1056-1064.e3 (2019).
57. Noecker, A. M. *et al.* StimVision v2: Examples and Applications in Subthalamic Deep Brain Stimulation for Parkinson's Disease. *Neuromodulation* **24**, 248–258 (2021).

58. Alho, E. J. L. *et al.* The Ansa Subthalamica: A Neglected Fiber Tract. *Movement Disorders* **35**, 75–80 (2020).

59. Horn, A. *et al.* Teaching NeuroImages: In vivo visualization of Edinger comb and Wilson pencils. *Neurology* **92**, e1663–e1664 (2019).

60. Hamani, C., Saint-Cyr, J. A., Fraser, J., Kaplitt, M. & Lozano, A. M. The subthalamic nucleus in the context of movement disorders. *Brain* **127**, 4–20 (2004).

61. Marani, E., Heida, T., Lakke, E. A. J. F. & Usunoff, K. G. The subthalamic nucleus. Part I: development, cytology, topography and connections. *Adv Anat Embryol Cell Biol* **198**, 1–113, vii (2008).

62. Yeh, F.-C. Population-based tract-to-region connectome of the human brain and its hierarchical topology. *Nat Commun* **13**, 4933 (2022).

63. Isaacs, B. R., Forstmann, B. U., Temel, Y. & Keuken, M. C. The Connectivity Fingerprint of the Human Frontal Cortex, Subthalamic Nucleus, and Striatum. *Frontiers in Neuroanatomy* **12**, (2018).

64. Middlebrooks, E. H. *et al.* Segmentation of the Globus Pallidus Internus Using Probabilistic Diffusion Tractography for Deep Brain Stimulation Targeting in Parkinson Disease. *AJNR Am J Neuroradiol* **107**, 64 (2018).

65. Yeh, F.-C. *et al.* Population-averaged atlas of the macroscale human structural connectome and its network topology. *NeuroImage* **178**, 57–68 (2018).

66. Van Essen, D. C. *et al.* The Human Connectome Project: A data acquisition perspective. *NeuroImage* **62**, 2222–2231 (2012).

67. Yeh, F.-C., Wedeen, V. J. & Tseng, W.-Y. I. Generalized q-Sampling Imaging. *IEEE Trans Med Imaging* **29**, 1626–1635 (2010).

68. Fedorov, A. *et al.* 3D Slicer as an Image Computing Platform for the Quantitative Imaging Network. *Magn Reson Imaging* **30**, 1323–1341 (2012).

69. Fonov, V. *et al.* Unbiased average age-appropriate atlases for pediatric studies. *NeuroImage* **54**, 313–327 (2011).

70. Fonov, V., Evans, A., McKinstry, R., Almli, C. & Collins, D. Unbiased nonlinear average age-appropriate brain templates from birth to adulthood. *NeuroImage* **47**, S102 (2009).

71. Alho, E. J. L. *et al.* High thickness histological sections as alternative to study the three-dimensional microscopic human sub-cortical neuroanatomy. *Brain Struct Funct* **223**, 1121–1132 (2018).

72. Alho, E. J. L. *et al.* The Ansa Subthalamica: A Neglected Fiber Tract. *Mov Disord* **35**, 75–80 (2020).

73. Parent, M. & Parent, A. The pallidofugal motor fiber system in primates. *Parkinsonism & Related Disorders* **10**, 203–211 (2004).

74. Mai, J. K., Majtanik, M. & Paxinos, G. *Atlas of the Human Brain*. (Elsevier Science, 2015).

75. Meola, A., Comert, A., Yeh, F.-C., Sivakanthan, S. & Fernandez-Miranda, J. C. The nondecussating pathway of the dentatorubrothalamic tract in humans: human connectome-based tractographic study and microdissection validation. *JNS* **124**, 1406–1412 (2016).

76. Nieuwenhuys, R., Voogd, J. & Van Huijzen, C. *The Human Central Nervous System*. (Springer Berlin Heidelberg, Berlin, Heidelberg, 2008). doi:10.1007/978-3-540-34686-9.

77. Coenen, V. A. *et al.* Tractographic description of major subcortical projection pathways passing the anterior limb of the internal capsule. Corticopetal organization of networks relevant for psychiatric disorders. *Neuroimage Clin* **25**, 102165 (2020).

78. Nieuwenhuys, R., Voogd, J. & van Huijzen, C. *The Human Central Nervous System*. (Springer Science & Business Media, Berlin, Heidelberg, 2013).

79. Coenen, V. A. *et al.* Surgical decision making for deep brain stimulation should not be based on aggregated normative data mining. *Brain Stimul* 1–5 (2019) doi:10.1016/j.brs.2019.07.014.

80. Ewert, S. *et al.* Toward defining deep brain stimulation targets in MNI space: A subcortical atlas based on multimodal MRI, histology and structural connectivity. *NeuroImage* **170**, 271–282 (2018).

81. Lavoie, B. & Parent, A. Pedunculopontine nucleus in the squirrel monkey: Projections to the basal ganglia as revealed by anterograde tract-tracing methods. *J. Comp. Neurol.* **344**, 210–231 (1994).

82. Kim, R., Nakano, K., Jayaraman, A. & Carpenter, M. B. Projections of the globus pallidus and adjacent structures: An autoradiographic study in the monkey. *J. Comp. Neurol.* **169**, 263–289 (1976).

83. Ding, S. *et al.* Comprehensive cellular-resolution atlas of the adult human brain. *J. Comp. Neurol.* **524**, 3127–3481 (2016).

84. Sébille, S. B. *et al.* Anatomical evidence for functional diversity in the mesencephalic locomotor region of primates. *NeuroImage* **147**, 66–78 (2017).

85. Gray, H. *Anatomy of the Human Body*. (Lea & Febiger, Philadelphia, 1918).

86. DeArmond, S. J. *Structure of the Human Brain: A Photographic Atlas*. (Oxford UP, 1989).

87. Humphrey, D. R., Gold, R. & Reed, D. J. Sizes, laminar and topographic origins of cortical projections to the major divisions of the red nucleus in the monkey. *J. Comp. Neurol.* **225**, 75–94 (1984).

88. Sarnat, H. B. Cerebellar networks and neuropathology of cerebellar developmental disorders. in *Handbook of Clinical Neurology* vol. 154 109–128 (Elsevier, 2018).

89. Courville, J. & Otabe, S. The rubro-olivary projection in the macaque: An experimental study with silver impregnation methods. *J. Comp. Neurol.* **158**, 479–491 (1974).

90. Kennedy, P. R. Corticospinal, rubrospinal and rubro-olivary projections: a unifying hypothesis. *Trends in Neurosciences* **13**, 474–479 (1990).

91. Butenko, K., Bahls, C., Schröder, M., Köhling, R. & van Rienen, U. OSS-DBS: Open-source simulation platform for deep brain stimulation with a comprehensive automated modeling. *PLoS Comp Biol* **16**, e1008023 (2020).

92. Predictors of Long-Term Outcome of Subthalamic Stimulation in Parkinson Disease - Cavallieri - 2021 - Annals of Neurology - Wiley Online Library. https://onlinelibrary.wiley.com/doi/10.1002/ana.25994.

93. Soulas, T., Sultan, S., Gurruchaga, J.-M., Palfi, S. & Fénelon, G. Depression and Coping as Predictors of Change After Deep Brain Stimulation in Parkinson's Disease. *World Neurosurgery* **75**, 525–532 (2011).

94. Bennett, D. A., Shannon, K. M., Beckett, L. A., Goetz, C. G. & Wilson, R. S. Metric properties of nurses' ratings of parkinsonian signs with a modified Unified Parkinson's Disease Rating Scale. *Neurology* **49**, 1580–1587 (1997).

95. Siderowf, A. *et al.* Test–Retest reliability of the Unified Parkinson's Disease Rating Scale in patients with early Parkinson's disease: Results from a multicenter clinical trial. *Movement Disorders* **17**, 758–763 (2002).

96. Metman, L. V. *et al.* Test–retest reliability of UPDRS-III, dyskinesia scales, and timed motor tests in patients with advanced Parkinson's disease: An argument against multiple baseline assessments. *Movement Disorders* **19**, 1079–1084 (2004).
