# Supplementary material for: Deep brain stimulation of symptom-specific networks in Parkinson’s disease
Source: Nat Commun. 2024 May 31;15:4662. doi: 10.1038/s41467-024-48731-1 (PMC11143329; doi:10.1038/s41467-024-48731-1)
Supplement: Supplementary file 3 — Description of Additional Supplementary Files [file 41467_2024_48731_MOESM3_ESM.pdf]

Description of Additional Supplementary Files

File Name: Supplementary Movie 1
Description: An example run of the Cleartune algorithm with four contrasting symptom profiles. Each panel shows how the algorithm would handle the symptom profile of a patient with maximal weighing of bradykinesia, rigidity, axial symptoms, tremor (clockwise). For instance, panel D shows a symptom profile of a typical tremor-dominant patient with high weighting of tremor and lower weighting of bradykinetic-rigid symptoms, which would favour settings that maximally target the tremor connections from primary motor cortex and cerebellum.
