# Supplementary material for: Deep brain stimulation of symptom-specific networks in Parkinson’s disease
Source: Nat Commun. 2024 May 31;15:4662. doi: 10.1038/s41467-024-48731-1 (PMC11143329; doi:10.1038/s41467-024-48731-1)
Supplement: Supplementary file 5 — Reporting Summary [file 41467_2024_48731_MOESM5_ESM.pdf]

nature portfolio

Corresponding author(s): Nanditha Rajamani

Last updated by author(s): Apr 15, 2024

Reporting Summary

Nature Portfolio wishes to improve the reproducibility of the work that we publish. This form provides structure for consistency and transparency in reporting. For further information on Nature Portfolio policies, see our Editorial Policies and the Editorial Policy Checklist.

Statistics

For all statistical analyses, confirm that the following items are present in the figure legend, table legend, main text, or Methods section.

n/a	Confirmed	
☐	☒	The exact sample size (*n*) for each experimental group/condition, given as a discrete number and unit of measurement
☐	☒	A statement on whether measurements were taken from distinct samples or whether the same sample was measured repeatedly
☐	☒	The statistical test(s) used AND whether they are one- or two-sided<br>*Only common tests should be described solely by name; describe more complex techniques in the Methods section.*
☐	☒	A description of all covariates tested
☐	☒	A description of any assumptions or corrections, such as tests of normality and adjustment for multiple comparisons
☐	☒	A full description of the statistical parameters including central tendency (e.g. means) or other basic estimates (e.g. regression coefficient) AND variation (e.g. standard deviation) or associated estimates of uncertainty (e.g. confidence intervals)
☐	☒	For null hypothesis testing, the test statistic (e.g. *F*, *t*, *r*) with confidence intervals, effect sizes, degrees of freedom and *P* value noted<br>*Give P values as exact values whenever suitable.*
☒	☐	For Bayesian analysis, information on the choice of priors and Markov chain Monte Carlo settings
☒	☐	For hierarchical and complex designs, identification of the appropriate level for tests and full reporting of outcomes
☐	☒	Estimates of effect sizes (e.g. Cohen's *d*, Pearson's *r*), indicating how they were calculated

Our web collection on statistics for biologists contains articles on many of the points above.

Software and code

Policy information about availability of computer code

Data collection	Microsoft Excel Version 16.65 & Matlab R2022b
Data analysis	MATLAB R2021b<br>Lead-DBS v2 (including tools: Lead-DBS Fiberfiltering, and adapted algorithms from SPM12, Advanced Normalization Tools, PaCER, Simbio, Fieldtrip): https://github.com/netstim/leaddbs, https://osf.io/bckuf/, https://github.com/netstim/SlicerNetstim<br>DSI Studio - 2022<br>3D Slicer Version 5.0.3<br>Mango Version 4.1<br>R-studio v4.2.1

For manuscripts utilizing custom algorithms or software that are central to the research but not yet described in published literature, software must be made available to editors and reviewers. We strongly encourage code deposition in a community repository (e.g. GitHub). See the Nature Portfolio guidelines for submitting code & software for further information.

Data

Policy information about availability of data

All manuscripts must include a data availability statement. This statement should provide the following information, where applicable:

- Accession codes, unique identifiers, or web links for publicly available datasets
- A description of any restrictions on data availability
- For clinical datasets or third party data, please ensure that the statement adheres to our policy

Cohort wise demographic and average of clinical information is available in Table 1. Patient imag-ing data cannot be openly shared due to data sharing and privacy regulation. However, they can be made available upon reasonable request to the corresponding primary investigators who acquired the data. The corresponding author and the principal investigator (NR and AH) commit to returning requests within a time frame of 30 days. A derivative of the E-fields, along with the corresponding patient outcomes are available in a dedicated repository within the Open Science Framework (OSF). Furthermore, the DBS Tractography Atlas, version 2.1, which was developed for the pre-sent study can be openly downloaded (https://github.com/netstim/DBS-Tractography-Atlas.git). The template for the development of the DBS tractography atlas is openly via DSI-Studio (HCP-1,065; https://brain.labsolver.org/hcp_template.html; fiber orientation maps at 1-mm resolution). DISTAL atlas, v 1.162 which was used for visualization of the basal ganglia nuclei is openly avail-able in the Lead DBS knowledge base and comes pre-installed in Lead DBS software.

Research involving human participants, their data, or biological material

Policy information about studies with human participants or human data. See also policy information about sex, gender (identity/presentation), and sexual orientation and race, ethnicity and racism.

Reporting on sex and gender	Proportions of self-identified sex information is available in the manuscript (table 1). Our present study does not perform extensive sex based analysis because prior literature does not suggest potential difference in symptom networks and circuits between different genders. However, we have used the sex information available to us as a categorical variable to determine if it significantly correlated with the therapeutic outcome.<br>Therefore, In the main analysis of the present study, we pool the sexes without using it as a covariate.
Reporting on race, ethnicity, or other socially relevant groupings	No other information such as race, ethnicity or other socially relevant groupings were available to us for the present analysis. Therefore, it was not used as a confounding variable for any analysis. Data for the original model (129 patients, termed "discovery cohort" in the manuscript) came from three different cohorts, and to investigate the center effects, we performed a small analysis to determine the significance of our results when controlling for "dataset" as the variable.
Population characteristics	Demographic information such as age, gender, and other clinically relevant factors (LEDD reduction, UPDRS baseline, UPDRS % improvement) have been provided in the main text of the manuscript (table 1). 232 patients from five centers who underwent bilateral STN-DBS for Parkinson's disease were retrospectively included in this study. 5 patients who also underwent bilateral STN-DBS for Parkinson's disease were prospectively included in the study.
Recruitment	232 were retrospectively included in this study. Prospective components of the study were included based on inclusion criteria as specified and approved by the institutional review board of University Würzburg in accordance with the Declaration of Helsinki. 5 patients were prospectively included in the study from the University of Würzburg, in accordance with the institutional review board, in accordance with the declaration of Helsinki
Ethics oversight	The study was approved by the institutional review board of Charité – Universitätsmedizin Berlin in accordance with the Declaration of Helsinki. Prospective components of the study were approved by the institutional review board of University Würzburg in accordance with the Declaration of Helsinki.

Note that full information on the approval of the study protocol must also be provided in the manuscript.

Field-specific reporting

Please select the one below that is the best fit for your research. If you are not sure, read the appropriate sections before making your selection.

☒ Life sciences ☐ Behavioural & social sciences ☐ Ecological, evolutionary & environmental sciences

For a reference copy of the document with all sections, see nature.com/documents/nr-reporting-summary-flat.pdf

Life sciences study design

All studies must disclose on these points even when the disclosure is negative.

Sample size	The sample size of the study is 237 patients, data for which was collected from 5 cohorts (Berlin = 51 patients, Wurzburg = 96 patients, Amsterdam = 34 patients, Beijing = 41 patients). Post hoc power analysis was performed. To get a power of 85% (power value commonly associated with significance), we require a sample size of n = 121 patients. Currently, our sample size is significantly above this, which puts us in a position to get a significant power of test.
Data exclusions	No data was excluded from the study

Replication	In order to determine the validity of our model we used data from 113 patients, from three cohorts. Our model generalized well on the entire cohort, and statistically significantly predicted the overall improvement in the outcomes for these patients (p = 0.001).
Randomization	Multiple iterations of k-10 analysis was performed with randomization to exclude the possibility of obtaining results due to chance.<br>In the prospective part of the study, the administration of cleartune and clinical settings were randomized, with the patient being blinded to the program that is administered.
Blinding	The authors were blinded to clinical data during imaging pre-processing and manual refinements to normalization and electrode localization. Cross validation were done in a model blinded manner - wherein the model was trained on a sample for patients and tested on a set of patients that were not seen by the model using a 5-,7- and 10- fold cross validation.<br>In the prospective component of the study, the patient is blinded to the program that is administered.

Reporting for specific materials, systems and methods

We require information from authors about some types of materials, experimental systems and methods used in many studies. Here, indicate whether each material, system or method listed is relevant to your study. If you are not sure if a list item applies to your research, read the appropriate section before selecting a response.

Materials & experimental systems

n/a	Involved in the study
☒	☐ Antibodies
☒	☐ Eukaryotic cell lines
☒	☐ Palaeontology and archaeology
☒	☐ Animals and other organisms
☒	☐ Clinical data
☒	☐ Dual use research of concern
☒	☐ Plants

Methods

n/a	Involved in the study
☒	☐ ChIP-seq
☒	☐ Flow cytometry
☐	☒ MRI-based neuroimaging

Plants

Seed stocks	N/A
Novel plant genotypes	N/A
Authentication	N/A

Magnetic resonance imaging

Experimental design

Design type	Retrospective, post-hoc analysis
Design specifications	N/A
Behavioral performance measures	N/A

Acquisition

Imaging type(s)	Structural
Field strength	1.5 T
Sequence & imaging parameters	Pre operative T1-weighted MRI, pre-operative T2-weighted MRI, Post-op CT and postop T1-weighted MRI
Area of acquisition	Whole brain

Diffusion MRI ☒ Used ☐ Not used

Parameters: multi-shell diffusion data, 31 Channels, b-values = 1000 s/mm2

Preprocessing

Preprocessing software	SPM, ANTs, LeadDBS v3, DSI-studio v3
Normalization	ANTs and SPM
Normalization template	ICBM 2009b Non linear space
Noise and artifact removal	Biasfield correction, Brain shift correction using coarse mask (Schoenecker 2008)
Volume censoring	N/A

Statistical modeling & inference

Model type and settings	Mass univariate approach, k-fold cross validation, permutation tests
Effect(s) tested	Fiber tractography correlated with clinical improvement (UPDRS-III) in Parkinson's Disease after STN-DBS.

Specify type of analysis: ☒ Whole brain ☐ ROI-based ☐ Both

Statistic type for inference (See Eklund et al. 2016)	k-fold cross validation; permutation tests; annova; t-test
Correction	FDR correction with an alpha < 0.05

Models & analysis

n/a	Involved in the study
☒	☐ Functional and/or effective connectivity
☒	☐ Graph analysis
☒	☐ Multivariate modeling or predictive analysis
